# Investigation of Multi-Ion Transport Properties in Cement Paste Based on a Multi-Scale Phase Evolution Model

**DOI:** 10.3390/ma19163479

**Published:** 2026-08-17

**Authors:** Zhuang Tian, Pan Zhang, Guanyan Xiao, Jin Xia, Weiliang Jin

**Affiliations:** Institute of Structural Engineering, Zhejiang University, Hangzhou 310058, China; 12312031@zju.edu.cn (Z.T.);

**Keywords:** cement paste, multi-scale microstructure, ion diffusion coefficient, phase evolution, chloride binding, calcium leaching, sulfate attack

## Abstract

**Highlights:**

**What are the main findings?**

- A multi-scale lattice diffusion–reaction framework integrating a phase evolution database enables efficient ion transport simulation.
- Multi-ion ingress generates highly ordered solid phase zonation featuring a characteristic double-step ettringite distribution.
- A simplified cracking criterion based on the capillary pore filling fraction captures the three-zone diffusion coefficient evolution.

**What are the implications of the main findings?**

- The framework provides a quantitative tool for durability assessment of concrete under complex chemical attack.
- The dual role of water-to-cement ratio offers guidance for durability design of marine concrete structures.
- The database approach significantly reduces computational cost in multi-scale reactive transport simulations.

**Abstract:**

Marine concrete structures are subjected to multiple aggressive ions that react with hydration products, driving dynamic phase evolution and altering ion transport pathways. This study develops a multi-scale lattice diffusion–reaction coupled framework grounded in a microstructural evolution model, incorporating a simplified analytical correction for the electrical double layer (EDL) effect. Validation against Poisson–Boltzmann numerical solutions across a pore size range of 1.5–50 nm confirms that the mean relative errors for monovalent, divalent, and trivalent ions remain within 10%. The phase evolution of cement paste under single-ion attack was simulated, and its impact on ion transport performance under multi-ion coupled ingress was systematically investigated. Under multi-ion attack, solid phases exhibit a highly ordered spatial zonation. Chloride ions completely displace monosulfate, forming a Friedel’s salt-enriched zone. Meanwhile, directly penetrating external sulfate generates a pronounced surface ettringite peak, while sulfate released from monosulfate decomposition in the Friedel’s salt zone induces secondary ettringite precipitation deeper within the material, producing a characteristic double-step ettringite distribution. A cracking criterion based on the critical capillary pore filling fraction captures the transition from pore filling to microcracking, yielding a three-zone profile for the relative diffusion coefficient. At 500 days of exposure, crystallization-induced microcracking triggers a more than 7-fold increase in surface relative diffusivity (w/c = 0.35). Furthermore, at 250 days, once cracking initiates, low water-to-cement ratio (w/c = 0.3) matrices display a higher relative diffusivity amplification factor of approximately 9, compared to approximately 6 for high water-to-cement ratio (w/c = 0.4) matrices. The established framework provides a quantitative tool for assessing the durability of concrete structures under complex chemical attack environments.

## 1. Introduction

Concrete is the most widely used construction material in the world. Its annual consumption is estimated at nearly 30 billion cubic meters [1]. With the continuous construction of major marine infrastructure such as cross-sea bridges and submarine tunnels [2], concrete structures are increasingly subjected to harsh marine environments, where aggressive ions, notably chloride and sulfate, cause severe durability deterioration and economic losses [3,4,5]. The ability of concrete to resist ion ingress is fundamentally controlled by the microstructure of cement paste [6], whose pore network spans from nano-scale gel pores of less than 10 nm to micro-scale capillary pores larger than 100 nm [7,8]. The mechanisms governing ion transport differ markedly across these scales. In capillary pores, transport is mainly controlled by topological parameters such as pore connectivity and tortuosity. In gel pores, an electrical double layer is formed at the charged surface of C-S-H, and this electrical double layer (EDL) significantly hinders ion motion. As a result, the diffusion coefficients of ions in gel pores can be more than one order of magnitude lower than those in bulk water [9,10]. Accurately capturing this EDL-induced nano-confinement effect remains a key challenge in predicting the macroscopic diffusion coefficient of cement-based materials.

The coexistence of multiple aggressive ions introduces complex chemical phase transformations that dynamically alter transport pathways [11,12]. Ingressing chloride reacts with monosulfate (AFm) to form Friedel’s salt, causing a modest volume expansion (~12%) and local pore filling [13,14,15]. Ingressing sulfate reacts with AFm to precipitate ettringite (AFt), producing a 135% volumetric expansion that generates crystallization pressure and triggers matrix microcracking once local tensile strength is exceeded [16,17]. Microstructural and durability investigations by Hussain et al. [18,19] further demonstrated how ettringite crystallization and solid-phase transformations govern pore structure degradation under sulfate and seawater exposure. Concurrently, calcium leaching induces CH dissolution and C-S-H decalcification, coarsening the pore network and increasing diffusivity [20,21,22]. These solid volume changes alter the porosity, while changes in pore structure modify the connectivity and tortuosity of transport pathways [23]. These comprehensive mechanistic investigations under various aggressive ions have progressively promoted the development of more refined numerical models to capture multi-ion coupled deterioration relationships.

Research on ion diffusivity prediction has evolved from macroscopic phenomenological models toward microscopic mechanistic formulations [24,25]. Garboczi and Bentz [26] pioneered effective diffusivity calculations based on CEMHYD3D digital microstructures. Subsequent studies by Zhang et al. [9], Achour et al. [27], Tong et al. [28], and Tian et al. [29] developed multi-scale homogenization methods and lattice diffusion networks based on general effective media theory. Furthermore, Friedmann et al. [30] and Qi et al. [31] applied Poisson–Boltzmann formulations to quantify electrical double layer confinement and electro-osmotic slip effects in nanopores. However, most existing microstructure-based transport models are restricted to static, undamaged pore networks, and cannot capture the dynamic evolution of transport pathways caused by chemical reactions and microcracking. In addition, directly coupling full numerical Poisson–Boltzmann solvers with multi-scale reactive transport algorithms remains computationally prohibitive, highlighting the need for an accurate yet practical analytical EDL correction.

To capture coupled chemical attack, integrated multi-ion modeling platforms have been developed. Elakneswaran and Ishida [32] established the DuCOM-PHREEQC coupling system, while Liu et al. [23] and Ohno and Maekawa [33] incorporated leaching and sulfate attack into multi-ion transport frameworks. However, existing platforms exhibit two major limitations. First, models such as DuCOM-PHREEQC primarily rely on macroscopic continuum formulations and empirical porosity–diffusivity relationships, lacking a physics-based multi-scale lattice transport bridge that explicitly connects C-S-H gel–pore electrical double layer confinement to paste-scale representative volume elements. Second, existing frameworks lack an efficient closed-loop phase database strategy capable of dynamically coupling local thermodynamic equilibrium zonation with real-time cross-scale transport parameter updates, resulting in heavy repetitive calculations that preclude iterative phase evolution simulations at practical time scales. Consequently, the staged characteristics, magnitude, and non-monotonic spatial evolution of relative diffusivity under microcracking damage cannot be systematically captured within a single model.

To resolve these challenges, this study establishes a multi-scale transport–reaction coupled framework by integrating thermodynamic equilibrium calculations with lattice diffusion theory, building upon a previously established multi-scale microstructure evolution model [23,34,35]. A three-level lattice diffusion network is constructed to calculate and transfer effective diffusivities from C-S-H gel to cement paste, incorporating a simplified analytical electrical double layer correction. A reaction module based on thermodynamic equilibrium principles is coupled with transport equations in a closed-loop manner, and an on-demand phase evolution database is developed to enable dynamic updates of local porosity and diffusion coefficients during multi-ion transport.

The primary contributions of this work are summarized as follows. First, a three-level lattice diffusion network incorporating a validated analytical electrical double layer correction is established, enabling physics-based cross-scale homogenization from gel pores to cement paste representative volume elements with a mean relative error below 10% against three-dimensional Poisson–Boltzmann solutions across 1.5 to 50 nm pores. Second, an on-demand phase evolution database strategy is developed to couple thermodynamic equilibrium calculations with real-time microstructural parameter updates, avoiding heavy repetitive calculations during transport modeling. Third, the spatial double-step ettringite zonation under multi-ion attack is systematically elucidated, and the non-monotonic spatial relative diffusivity evolution characterized by a surface cracking surge, a subsurface pore-filling drop, and an unperturbed deep baseline is revealed under microcracking. Together, these contributions form a closed-loop framework capable of predicting the coupled evolution of ion transport, phase composition, and microcracking in cement paste under multi-ion attack, offering a computationally efficient tool for durability assessment.

## 2. Model Methodology

The multi-scale microstructure evolution model developed by previous researchers [34,35] was adopted as the fundamental geometric framework for ion diffusion coefficient calculation. In this model, hardened cement paste is divided into three characteristic scale levels, and a parameter transfer strategy is employed to achieve cross-scale connection. All model construction and coding work in this study were carried out on the MATLAB R2024a platform.

### 2.1. Representative Volume Element (RVE) Construction at Three Scale Levels

The ion transport performance of cement-based materials is governed by their complex phases spanning from nanometer to micrometer scales [1,7]. At the nano-scale, the gel pore network formed through the packing of C-S-H gel particles directly determines the local transport capacity of the hydration product phase [7,8]. At the sub-micron scale, the small capillary pores in the outer C-S-H layer serve as the key transport bridge connecting hydration products and large capillary pores [35]. At the micro-scale, the distribution of various phases and large capillary pores dominates the macroscopic diffusion performance of cement paste [34]. Given these multi-scale characteristics, ion transport behavior cannot be accurately described by a single-scale model [36]. A multi-scale modelling strategy was therefore adopted, in which the microstructure was divided into the C-S-H gel scale (Level 1), hydration product scale (Level 2), and cement paste scale (Level 3), with independent reconstruction and parameter transfer performed at each scale.

#### 2.1.1. C-S-H Gel Scale (Level 1)

At the C-S-H gel scale, three-dimensional RVEs of low-density C-S-H (LD C-S-H, gel porosity 37%) and high-density C-S-H (HD C-S-H, gel porosity 24%) were generated using a hard core–soft shell (HCSS) packing algorithm [37,38]. The basic structural units of both types of C-S-H gel are colloidal particles of approximately 5 nm in diameter [7], with a hard-core-to-soft-shell size ratio of 2:3. Particle placement was continued until the target porosity was reached. The RVE had a side length of 100 nm, a resolution of 0.5 nm/voxel (201^3^ voxel grid), and periodic boundary conditions were applied.

**Figure 1:**
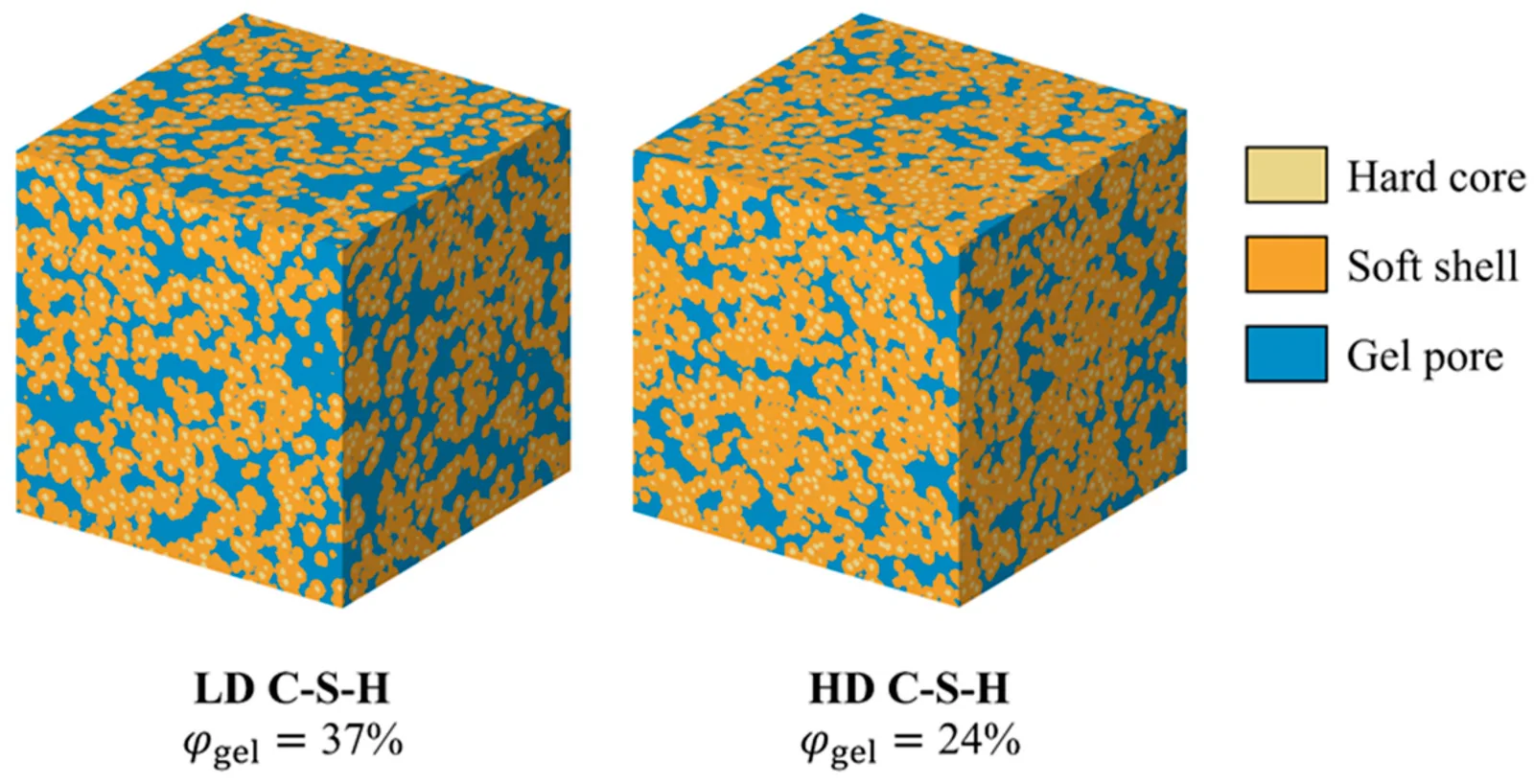
Three-dimensional rendered RVE images at the C-S-H gel scale.

Typical three-dimensional rendered RVE images of C-S-H are presented in Figure 1. In LD C-S-H, the relatively high gel porosity results in loose packing among colloidal particles, a broad gel pore distribution (pore diameters approximately 1–10 nm), and good connectivity. HD C-S-H, with a gel porosity of only 24%, exhibits tightly packed colloidal particles and gel pores highly concentrated in the 1–4 nm range. Most of the gel pore sizes fall within the region of significant EDL overlap (below approximately 2–3 nm), where the ion concentration distribution is strongly regulated by the wall electrostatic field. This fundamentally explains why the inner hydration products contribute minimally to overall transport, despite their considerable volume fraction [27].

#### 2.1.2. Hydration Product Scale (Level 2)

At the hydration product scale, two representative units (the outer C-S-H layer and the inner hydration products) were constructed separately. The outer C-S-H layer was modelled as a two-phase composite system consisting of LD C-S-H gel and small capillary pores (12–100 nm). Following the modelling strategy of Ma et al. [36], a small number of LD C-S-H nuclei were randomly generated within the RVE, and the aggregation and growth of LD C-S-H were subsequently simulated by continuously adding new spheres. The RVE was constructed with a side length of 500 nm and a resolution of 2.5 nm/voxel (201^3^ voxel grid). The small capillary porosity $\varphi_{\mathrm{ocsh}}$ was related to the hydration degree $\alpha_{\mathrm{RVE}}$ and the initial water-cement ratio w/c through Equation (1) [36].

(1) $$\varphi_{\mathrm{ocsh}}=\frac{0.63w/c-0.226\alpha_{\mathrm{RVE}}}{0.852w/c-0.09\alpha_{\mathrm{RVE}}}$$

**Figure 2:**
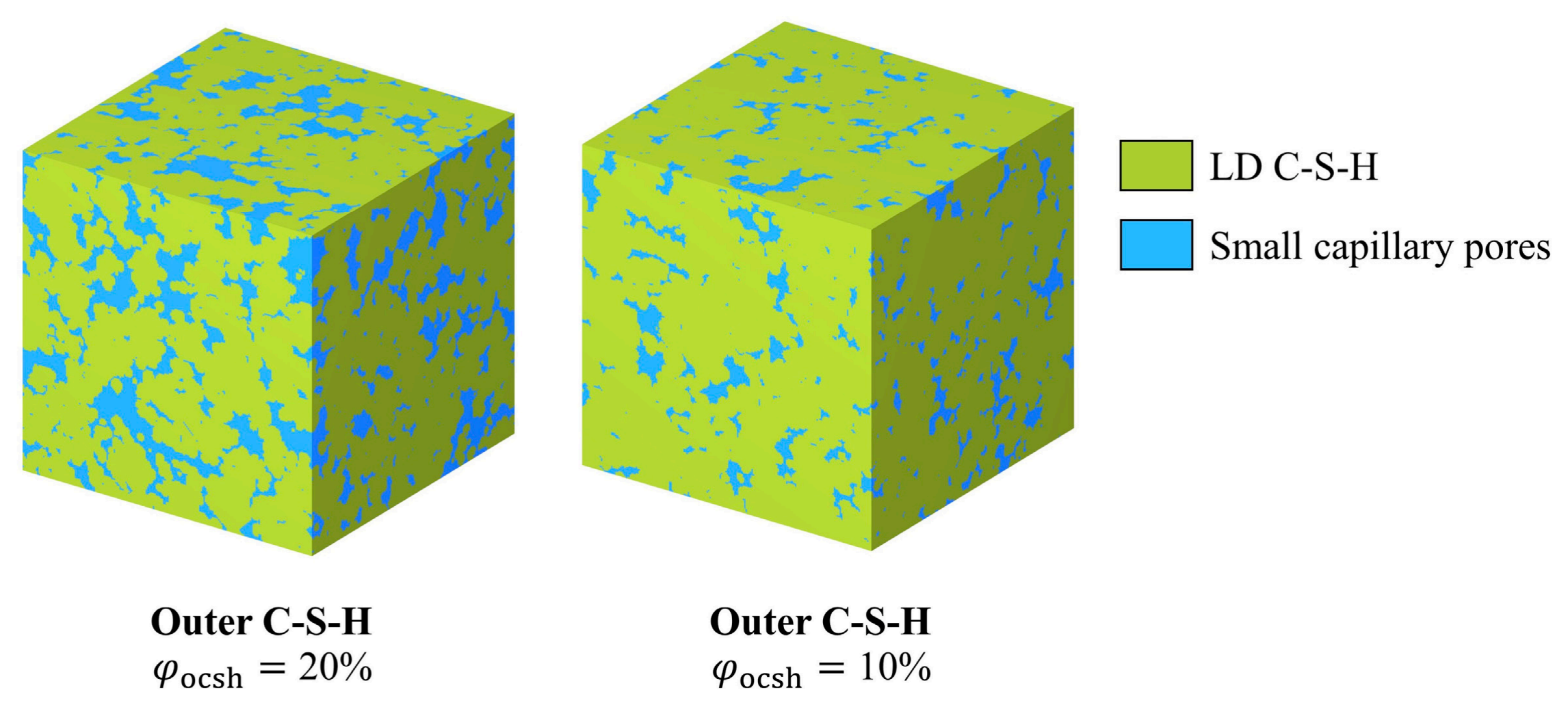
Three-dimensional rendered RVE images of the outer C-S-H layer under different small capillary porosities.

Figure 2 presents the typical three-dimensional microstructures of the outer C-S-H layer at different small capillary porosities. When $\varphi_{\mathrm{ocsh}}$ is relatively high (approximately 10–30%), the LD C-S-H particles are loosely clustered and the small capillary pore network remains highly connected. These features are consistent with the morphological characteristics of the outer C-S-H layer that has not yet been fully densified during early hydration. As $\varphi_{\mathrm{ocsh}}$ gradually decreases, the LD C-S-H particles become more densely packed, the small capillary pore network shrinks significantly, and the proportion of isolated pores increases. When $\varphi_{\mathrm{ocsh}}$ drops further to below 10%, the structure becomes considerably dense, with only a small number of isolated or semi-connected small capillary pores retained. This packing-density-driven topological evolution of the pore network constitutes the geometric origin of the dynamic variation in the outer C-S-H layer diffusion coefficient with hydration degree [35,36].

**Figure 3:**
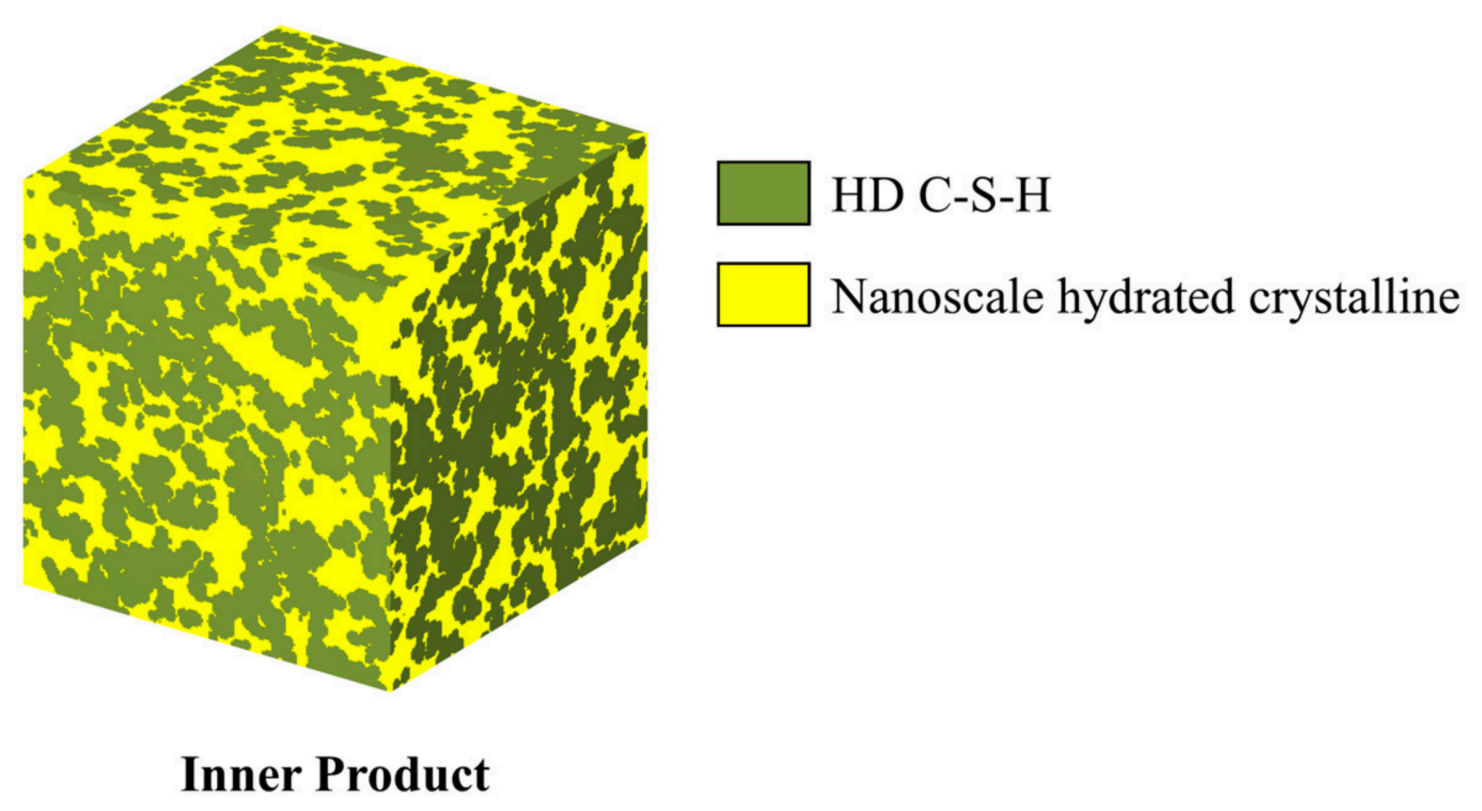
Three-dimensional rendered RVE images of the inner product layer.

The inner hydration products form a dense mixed system within the original boundary of the unhydrated cement clinker particles and are composed of HD C-S-H and nano-scale hydration crystalline phases. Typical three-dimensional rendered RVE images of inner product are presented in Figure 3. The RVE was constructed with a side length of 5000 nm and a resolution of 25 nm/voxel, and the characteristic diameter of HD C-S-H was taken as 200 nm according to Jennings [39]. The volume fraction of the crystalline phase was determined from the stoichiometric relationships of the cement mineral composition [35]. Since the gel pore connectivity of HD C-S-H is already near zero, and the sparse gel pore channels are further blocked by the embedded non-diffusible crystalline phase particles, the contribution of the inner hydration products to macroscopic ion transport is extremely limited [27]. In the subsequent diffusion coefficient calculation at the cement paste scale, these products were treated as a low-diffusivity phase.

#### 2.1.3. Cement Paste Scale (Level 3)

At the cement paste scale, the RVE was constructed with a side length of 100 μm and a resolution of 0.5 μm/voxel (201^3^ voxel grid), and periodic boundary conditions were applied. The particle size distribution of cement was described by the Rosin–Rammler function [40].

(2) $$P_{V}\left(d\right)=1-\exp\left(-b\times d^{n}\right)$$

where $P_{V}\left(d\right)$ is the cumulative volume distribution function of cement particles, d is the cement particle diameter, and b and n are constants related to the mean particle size and dispersion degree of the cement, respectively. Referring to a typical Portland cement with a specific surface area of approximately 350 m^2^/kg, the values b=0.034 and n=1.09 were adopted [41]. The cement used in this study is a reference cement conforming to the Chinese national standard for admixture testing, and the mineralogical composition per unit mass is listed in Table 1. The minimum and maximum particle diameters were set at 2 μm and 50 μm, respectively. The initial particle placement was performed using a Monte Carlo random packing algorithm under periodic boundary conditions, with particles introduced in descending order of size to improve the packing success rate at high volume fractions [41].

**Figure 4:**
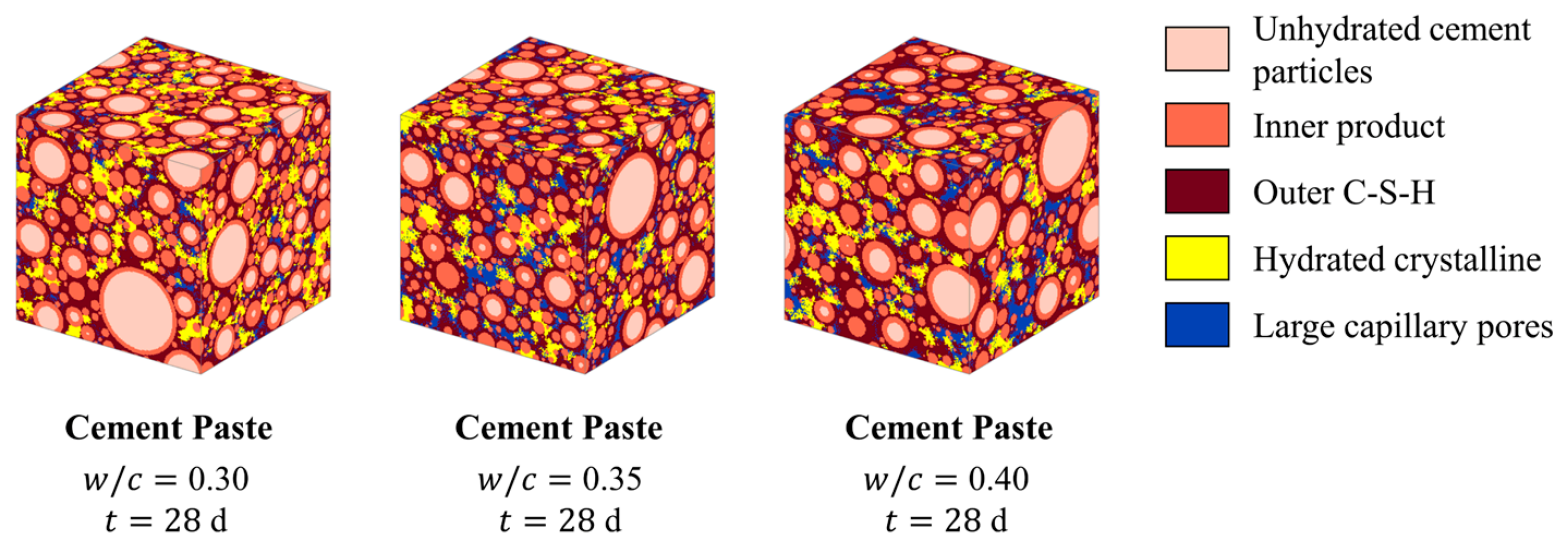
Typical three-dimensional RVEs of cement paste layer for three water-to-cement ratios.

The microstructural evolution was driven by the water-transport-based hydration kinetics model proposed by Rahimi-Aghdam et al. [42]. The core assumption of this model is that water permeates through the progressively thickening C-S-H gel layer surrounding the unhydrated cement particle toward the inner core, and this mass transport process constitutes the decisive factor controlling the medium- and long-term hydration rate [43]. Following the approach of Duan et al. [35], the model was extended to polydisperse particle systems, and bidirectional coupling between hydration kinetics and microstructure was achieved through feedback of the free surface area of the particles. The RVEs of cement paste with three water-to-cement ratios of 0.3, 0.35, and 0.4 were generated under saturated conditions at 25 °C for a hydration period of 28 days, as illustrated in Figure 4. During the growth of hydration products, higher weighting factors were assigned to sites closer to the cement particle surface and those surrounded by more open pore space to reproduce the non-uniform morphology of outer hydration products. As a result, the products were preferentially deposited at these sites, naturally capturing the realistic morphological sequence of layered and plate-like growth at the early stage, a dense interior with a sparse exterior at the middle stage, and the retention of dead-end and isolated pores at the late stage.

The microstructure model was established based on the following fundamental assumptions. The RVEs at each scale are statistically homogeneous and isotropic, and the overall transport performance of the material at that scale can be represented by a local volume [28]. When properties are transferred from a lower scale to a higher scale, the lower-scale components are treated as homogeneous continuous media, with their internal complex pore structure characterized by a single effective diffusion coefficient [27]. The ion transport process is assumed to be at steady state, and local equilibrium is maintained in the physicochemical interaction between ions and pore walls [35]. Cement particles are treated as spherical, and the secondary influence of irregular particle morphology on transport pathways is neglected [26]. The hydration reactions of different clinker components are assumed to proceed independently without mutual interference, and the types and relative amounts of hydration products are determined by stoichiometric relationships [43]. The paste is assumed to remain saturated and at constant temperature (25 °C), and any effects of moisture transport or thermal gradients are not considered.

### 2.2. Multi-Scale Calculation Method for Ion Diffusion Coefficient

#### 2.2.1. Lattice Diffusion Network Model

A lattice diffusion network method [26,28,44] was employed to calculate the effective diffusion coefficients of RVEs at each level. In this method, each voxel of a diffusible phase in the three-dimensional voxelized microstructure is treated as a lattice node, and mass transport between nodes is governed by Fick’s first law. The calculation is performed under steady-state diffusion without considering ion binding effects. Compared with the finite element method, the lattice diffusion network model can directly handle the spatially heterogeneous distribution of diffusion coefficients of different phases.

**Figure 5:**
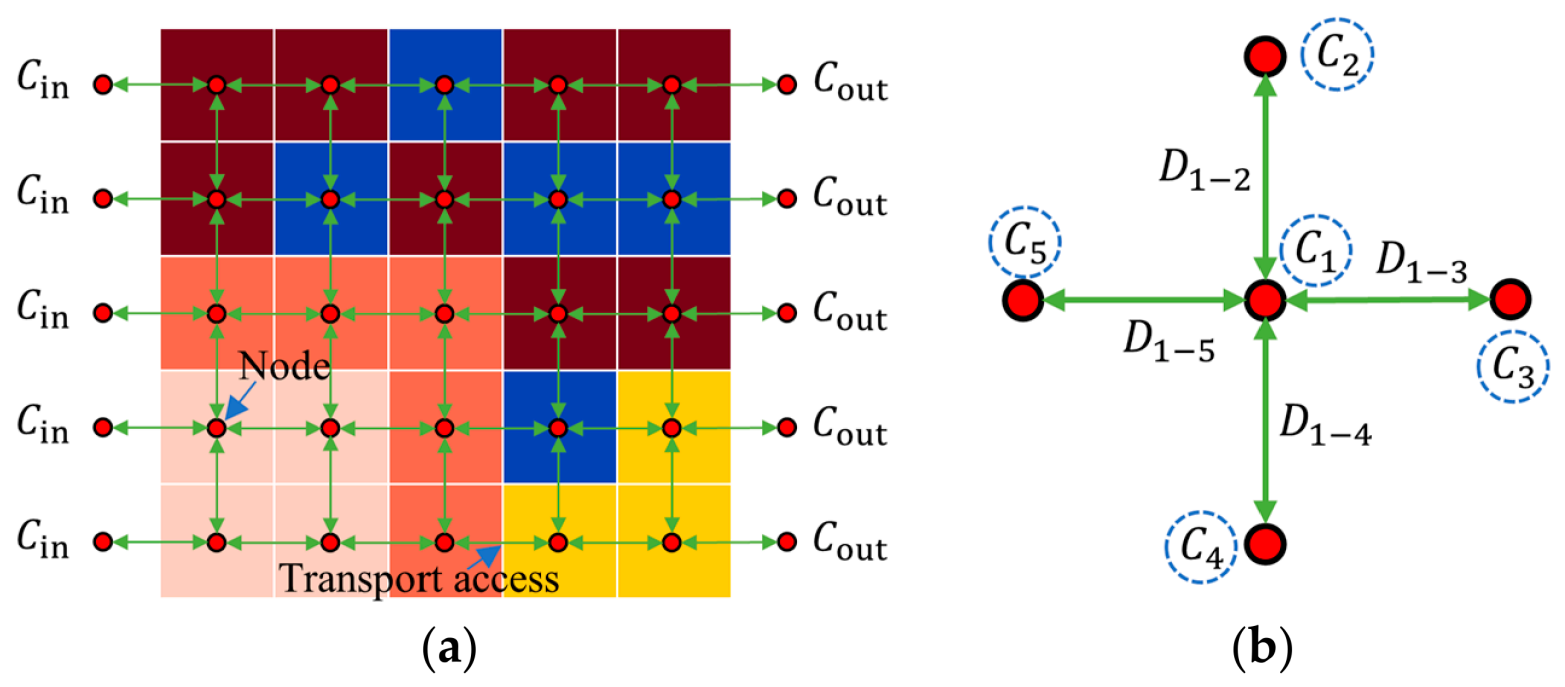
Schematic of microstructure voxelization and lattice diffusion network conversion: (**a**) multi-phase microstructure slice; (**b**) corresponding lattice diffusion network.

The conversion from a three-dimensional voxelized microstructure to a lattice diffusion network is illustrated schematically in Figure 5. In the voxelized image, each voxel is classified as either a diffusible phase or a non-diffusible solid phase. In the corresponding lattice network, every voxel is abstracted as a node. Each node is connected only to its six face-adjacent neighbors, meaning that mass exchange occurs exclusively between directly adjacent voxels. Nodes representing diffusible phases are assigned diffusion coefficients according to their phase type, whereas nodes representing non-diffusible solids are assigned a diffusion coefficient of zero. Further refinements to the diffusion coefficients based on local geometric characteristics and EDL effects are introduced in the following subsection. The resulting lattice network thus captures the topology of the pore space and provides the geometric basis for computing the effective diffusion coefficient.

Under steady-state conditions, the total flux entering any internal node i from its adjacent nodes remains zero. For a three-dimensional 6-neighbor configuration, the mass conservation equation is given by

(3) $$\sum_{j\in \Omega_{i}}D_{i-j}\left(c_{j}-c_{i}\right)=0$$

where $\Omega_{i}$ is the set of nodes directly face-adjacent to node i (up to 6 nodes), and $c_{i}$ and $c_{j}$ are the ion concentrations at nodes *i* and *j*, respectively. The interfacial diffusion coefficient $D_{i-j}$ connecting nodes *i* and *j* is obtained by the harmonic mean [26].

(4) $$D_{i-j}=\frac{2}{\frac{1}{D_{i}}+\frac{1}{D_{j}}}$$

where $D_{i}$ and $D_{j}$ are the intrinsic diffusion coefficients of adjacent nodes i and j, respectively. The physical implication of the harmonic mean is interpreted as follows. When the diffusion coefficient of one of the two adjacent nodes is relatively small, for example, a node located near a pore wall whose local diffusion coefficient is strongly suppressed by the EDL effect, the equivalent diffusion coefficient at the interface is dominated by the higher transport resistance on that side. Consequently, $D_{i-j}$ is made closer to the smaller of the two values [26].

Fixed concentration boundary conditions were imposed on a pair of opposing faces of the RVE, namely the x=0 and x=L planes. The inlet face concentration was set to $C_{\mathrm{in}}=10$ mol/m^3^, and the outlet face concentration was set to $C_{\mathrm{out}}=0$ mol/m^3^. The remaining four side faces were set as zero-flux boundary conditions. The conjugate gradient method [30] was adopted for the iterative solution of the above linear system of equations, and a convergence tolerance of 10^−8^ was applied. Once the diffusion reached a steady state, the effective diffusion coefficient $D_{\mathrm{eff}}$ of the RVE was calculated from the total flux Q at the outlet face.

(5) $$D_{\mathrm{eff}}=\frac{Q}{A}\cdot \frac{L}{C_{\mathrm{in}}-C_{\mathrm{out}}}$$

where L is the length of the RVE along the diffusion direction, and A is the area of the outlet face. For a cubic RVE with side length L, $A=L^{2}$. Full consistency between Equation (5) and the macroscopic expression of Fick’s first law is observed [28].

#### 2.2.2. Analytical Correction for the Electrical Double Layer Effect

In the nano-scale pores of cement-based materials, ion transport behavior differs substantially from that in bulk water. According to the electrical double layer (EDL) theory, the diffusion coefficients of ions decrease sharply in regions closer to the pore wall. In nano-scale gel pores (smaller than approximately 10 nm), the deprotonation of silanol groups (≡SiOH) on the C-S-H surface imparts a negative charge to the pore wall [45]. Cations (Ca^2+^, Na^+^, K^+^) in the pore solution accumulate near the solid–liquid interface to form a Stern layer. However, owing to strong electrostatic binding and surface chemical adsorption, their effective diffusion coefficients approach zero in this layer. Anions such as Cl^−^ are electrostatically repelled, and their local concentration is significantly lower than that in the bulk pore solution [30], with the effective diffusion coefficient similarly approaching zero. When the pore size is comparable to the Debye length (pore diameter approximately 2–10 nm), the EDLs originating from opposing pore walls overlap, and the diffusion coefficients of most ions within the pore become significantly lower than those in bulk water [9].

A rigorous treatment of the EDL effect requires solving the Poisson–Boltzmann (PB) equation to obtain the spatial distribution of the electrostatic potential and ion concentrations within each pore, which would then be coupled to the lattice diffusion calculation [9]. Such an approach, however, would be computationally prohibitive for the multi-scale RVE framework adopted in this study, given the vast number of gel pores and the iterative nature of the phase evolution simulations. An analytical correction strategy is therefore introduced to capture the dominant influence of the EDL on ion diffusivity at an acceptable computational cost. The fundamental idea is to replace the full PB solution with a parametric function that estimates the local reduction in diffusivity based solely on the geometric position of each voxel relative to the nearest solid wall. This strategy necessarily introduces simplifications, and its accuracy is intrinsically limited by the assumptions described below.

According to the Gouy–Chapman–Stern model, the ion distribution and electrostatic potential field near a charged solid surface are governed by the PB equation [46]. For a one-dimensional planar interface, the electrostatic potential $\psi \left(x\right)$ within the diffuse layer decays with the distance *x* from the outer boundary of the Stern layer as

(6) $$\psi \left(x\right)=\psi_{0}\exp\left(-\frac{x}{\lambda_{D}}\right)$$

where $\psi_{0}$ is the reference potential at the outer boundary of the Stern layer and $\lambda_{D}$ is the Debye length, which characterizes the attenuation thickness of the electrostatic potential in the diffuse layer. Based on this exponential decay characteristic, the local ion diffusion coefficient $D\left(d,R_{c}\right)$ for each gel–pore voxel is assigned in a spatially differentiated manner as a function of the shortest distance d from the voxel to the nearest solid wall and the characteristic pore radius $R_{c}$, with the latter determined by the maximum inscribed sphere algorithm [47].

(7) $$D\left(d,R_{c}\right)=D_{\mathrm{bulk}}\left[1-\exp\left(-\frac{d-\delta }{\kappa \lambda_{D}}\right)\right]\cdot \left[1-\exp\left(-\frac{2R_{c}-d-\delta }{\kappa \lambda_{D}}\right)\right]$$

The parameters in Equation (7) are defined as follows. $D_{\mathrm{bulk}}$ is the self-diffusion coefficient of the ion in bulk water at 25 °C, which is an intrinsic property of each ionic species and constitutes the primary source of differentiation among ions in the model. d is the shortest Euclidean distance from the voxel centre to the nearest solid wall, obtained via a three-dimensional Euclidean distance transform [48]. δ is the thickness of the Stern layer, within which ions are immobilised by strong electrostatic binding; a uniform value of 0.8 nm is adopted for all ionic species [9]. $\lambda_{D}$ is the Debye length, which characterises the attenuation thickness of the diffuse layer; based on the typical ionic strength of cement pore solution (0.3–0.5 mol/L), a uniform value of 0.6 nm is adopted for all ions [9]. κ is a charge-related confinement factor. While δ and $\lambda_{D}$ are taken as identical for all ions, the factor κ in the denominator of Equation (7) serves as a charge-dependent confinement parameter.

In this analytical formulation, two simplifying assumptions are introduced. First, a major simplifying assumption is made regarding the electrostatic field effects. In nano-scale gel pores, the diffusivity of all ions—whether cations or anions—is suppressed near the charged pore wall due to electrostatic interactions and the finite size of hydrated ions. Rather than resolving the distinct behaviors of cations and anions, which would require additional ion-specific parameters and substantially higher computational cost, the electric field is pragmatically assumed to universally suppress the local diffusivity of all ionic species. Consequently, the spatial restriction depends symmetrically on the charge magnitude $\left|Z_{k}\right|$ rather than the signed charge $Z_{k}$. It should be acknowledged that this simplification is clearly inaccurate at the individual pore level, where cations and anions exhibit fundamentally different near-wall concentration and mobility profiles. Nevertheless, at the cement paste scale, the computational results presented below demonstrate that the predicted effective diffusion coefficients for major ions agree reasonably well with reported values, and the phase evolution results exhibit satisfactory agreement with experimental data (see Section 3.4). This indicates that the simplified treatment captures the dominant effects of EDL confinement at the macroscopic level without introducing systematic errors in the upscaled transport properties. Second, κ is a charge-dependent empirical parameter whose values are calibrated against the full PB-steric cylinder-shaped pore solution (detailed in Section 2.2.3). Monovalent ions ($\left|Z_{k}\right|$ = 1) are assigned a baseline value of κ = 1.0. For multivalent ions experiencing stronger electrostatic interactions and larger hydration shells, smaller denominator values are assigned, κ = 0.6 for divalent ions ($\left|Z_{k}\right|$ = 2) and κ = 0.4 for trivalent ions ($\left|Z_{k}\right|$ = 3). A smaller κ value accelerates the exponential decay near the wall, thereby contracting the accessible core of the transport channel.

The two bracketed terms in Equation (7) capture the dual confinement exerted by opposing pore walls. The first term represents unilateral decay from the nearest wall, while the second term accounts for restriction from the opposing wall when the pore diameter is small. Although Equation (7) provides a computationally efficient surrogate for multi-scale calculations, its physical rationale and quantitative fidelity must be rigorously verified. The physical reasonableness of Equation (7) is demonstrated by its strict compliance with fundamental physical boundary conditions. Within the Stern layer (d≤δ or $2R_{c}-d\leq \delta$), the first or second bracketed term vanishes, forcing $D\left(d,R_{c}\right)=0$, which strictly satisfies the zero-velocity, complete immobilization boundary condition at solid interfaces. Conversely, in the center of large capillary pores ($d\gg \delta +\lambda_{D}$ and $2R_{c}-d\gg \delta +\lambda_{D}$), both bracketed terms approach unity, allowing the local diffusivity to smoothly recover to its bulk water value $D_{\mathrm{bulk}}$.

The parameter values adopted for the major ionic species are summarized in Table 2. It should be noted that this analytical treatment provides only a first-order approximation of the EDL effect. The actual diffusivity of a confined ion depends on additional factors such as the hydration shell structure and specific ion–surface interactions, which are not explicitly resolved here. These simplifications are considered acceptable within the scope of this study, which prioritizes computational efficiency and cross-scale coupling over the precise resolution of individual ion–surface interactions. A detailed discussion on the physical justification of Equation (7), its handling of charge valence, and its quantitative validation against the full Poisson–Boltzmann model are presented in Section 2.2.3.

Within the adopted framework, Equation (7) is applied to assign a spatially heterogeneous diffusion coefficient to every voxel in each gel pore. After the voxel-level diffusivities are assigned, the lattice diffusion network model described in Section 2.2.1 is executed to compute the effective diffusion coefficient of the RVE at that scale. This completes the integration of the EDL correction into the multi-scale calculation chain. Following the stepwise parameter transfer strategy from lower to higher scales, ten parallel RVEs were generated at each scale and the averaged effective diffusion coefficient was taken as the result.

At the C-S-H gel scale (Level 1), lattice diffusion calculations were performed on the RVEs of LD C-S-H and HD C-S-H. The gel pore voxels were assigned spatially heterogeneous local diffusion coefficients via Equation (7), while the C-S-H solid particles were treated as non-diffusible. The effective diffusion coefficients $D_{\mathrm{LD},\;i}$ and $D_{\mathrm{HD},\;i}$ were thus obtained.

At the hydration product scale, lattice diffusion calculations were conducted separately for the outer C-S-H layer and the inner hydration products (Level 2). The outer C-S-H layer contains LD C-S-H gel, to which $D_{\mathrm{LD},i}$ was assigned, and small capillary pores, whose local diffusion coefficients were corrected by the EDL via Equation (7). The inner hydration products consist of HD C-S-H assigned $D_{\mathrm{HD},i}$ and nano-scale crystalline phases treated as non-diffusible. The effective coefficients $D_{\mathrm{ocsh},i}$ and $D_{\mathrm{inner},i}$ were obtained.

At the cement paste scale (Level 3), differentiated diffusion coefficients were assigned to all phases. Large capillary pores, with diameters far exceeding the Debye length, were assigned $D_{\mathrm{bulk},i}$ with negligible EDL correction. The outer C-S-H layer was assigned $D_{\mathrm{ocsh},i}$, and the inner hydration products $D_{\mathrm{inner},\;i}$. Non-diffusible phases, including unhydrated cores and micro-scale crystalline phases, were set to zero. The macroscopic effective diffusion coefficient $D_{\mathrm{paste},i}$ of the cement paste was calculated from the lattice network at this scale.

**Figure 6:**
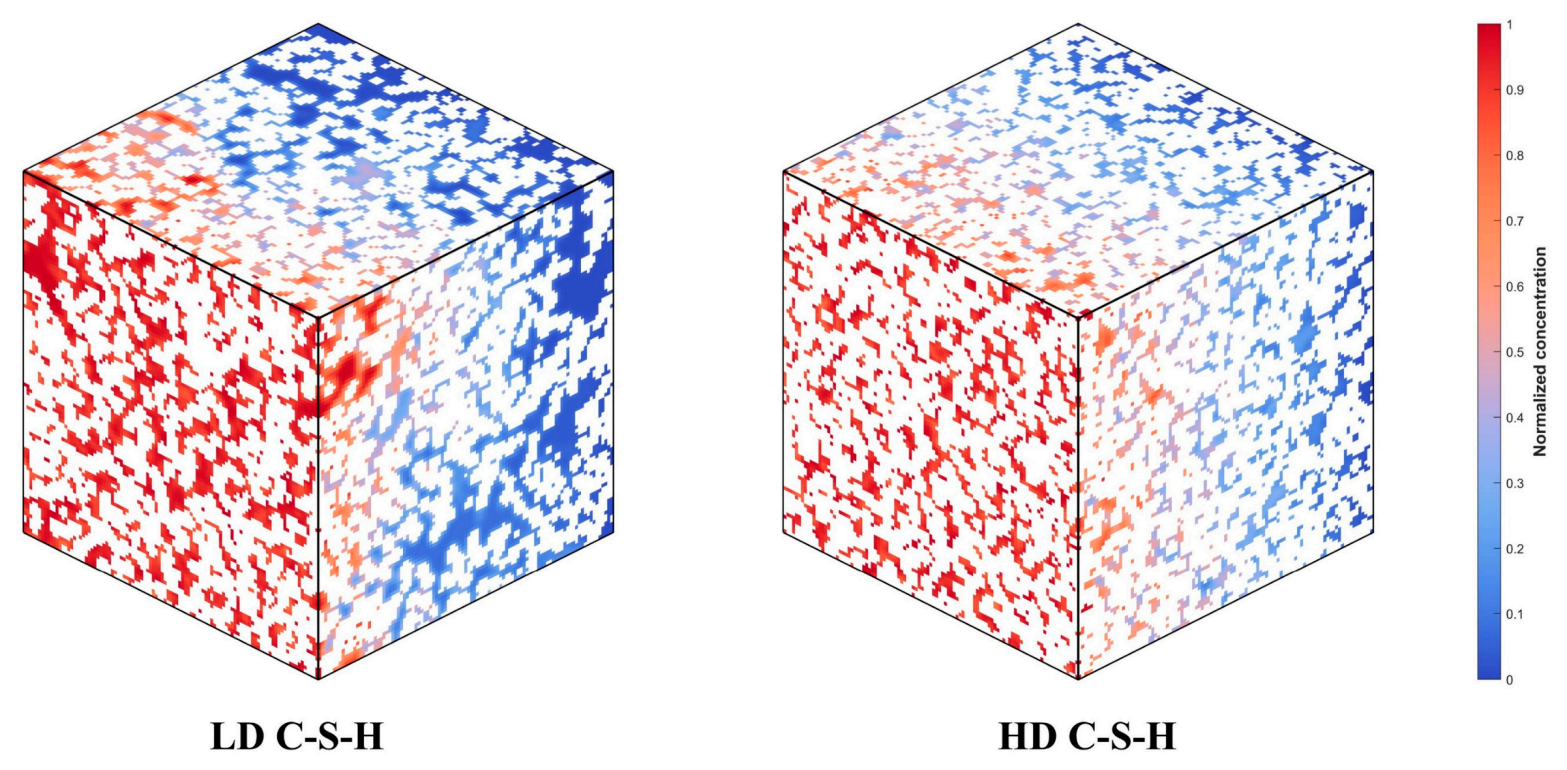
Distribution of chloride ions during steady-state diffusion at the C-S-H level.

Taking chloride as an example, the steady-state ion concentration distribution within the C-S-H gel RVEs is shown in Figure 6. In LD C-S-H, the gel pores form a reasonably connected network that enables ion migration. However, the local diffusivity near pore walls is strongly suppressed by the EDL effect, so ion transport preferentially follows the high-diffusivity channels at pore centers. In HD C-S-H, the gel pores are concentrated in the 1–4 nm range, with most of the pore space falling within the EDL-overlap region. As a result, the overall diffusion coefficient of HD C-S-H is considerably lower than that of LD C-S-H.

The computed diffusion coefficients for cement pastes with three water-to-cement ratios (0.30, 0.35, 0.40) after 28 days of saturated curing are summarized in Table 3. The diffusion coefficients increase significantly with increasing w/c, a trend directly attributable to the higher capillary porosity and improved pore connectivity. Differences among ionic species are primarily governed by their bulk self-diffusion coefficients: ions with higher $D_{\mathrm{bulk}}$ (e.g., OH^−^, 5.26 × 10^−9^ m^2^/s) exhibit correspondingly higher diffusion coefficients in the paste, whereas those with low $D_{\mathrm{bulk}}$ (e.g., Al^3+^, 0.54 × 10^−9^ m^2^/s) diffuse the slowest. In addition, multivalent ions (Ca^2+^, Al^3+^, SO_4_^2−^) receive stronger EDL suppression due to their smaller κ values (κ < 1), which further widens the diffusion coefficient gaps among ions. The calculated values are consistent with previously reported results [27,49].

To facilitate the comparison of the effects of different chemical attacks on ion diffusion, the relative diffusion coefficient $D/D_{0}$ was uniformly adopted in the subsequent analysis, where $D_{0}$ is the initial effective diffusion coefficient of the unattacked reference cement paste and D is the coefficient at a given state of attack. Quantitative analysis of the evolution of $D/D_{0}$ can directly reveal the direction, magnitude, and staged characteristics of the influence of different chemical degradation mechanisms on ion transport.

#### 2.2.3. Validation of the Analytical EDL Correction

**Figure 7:**
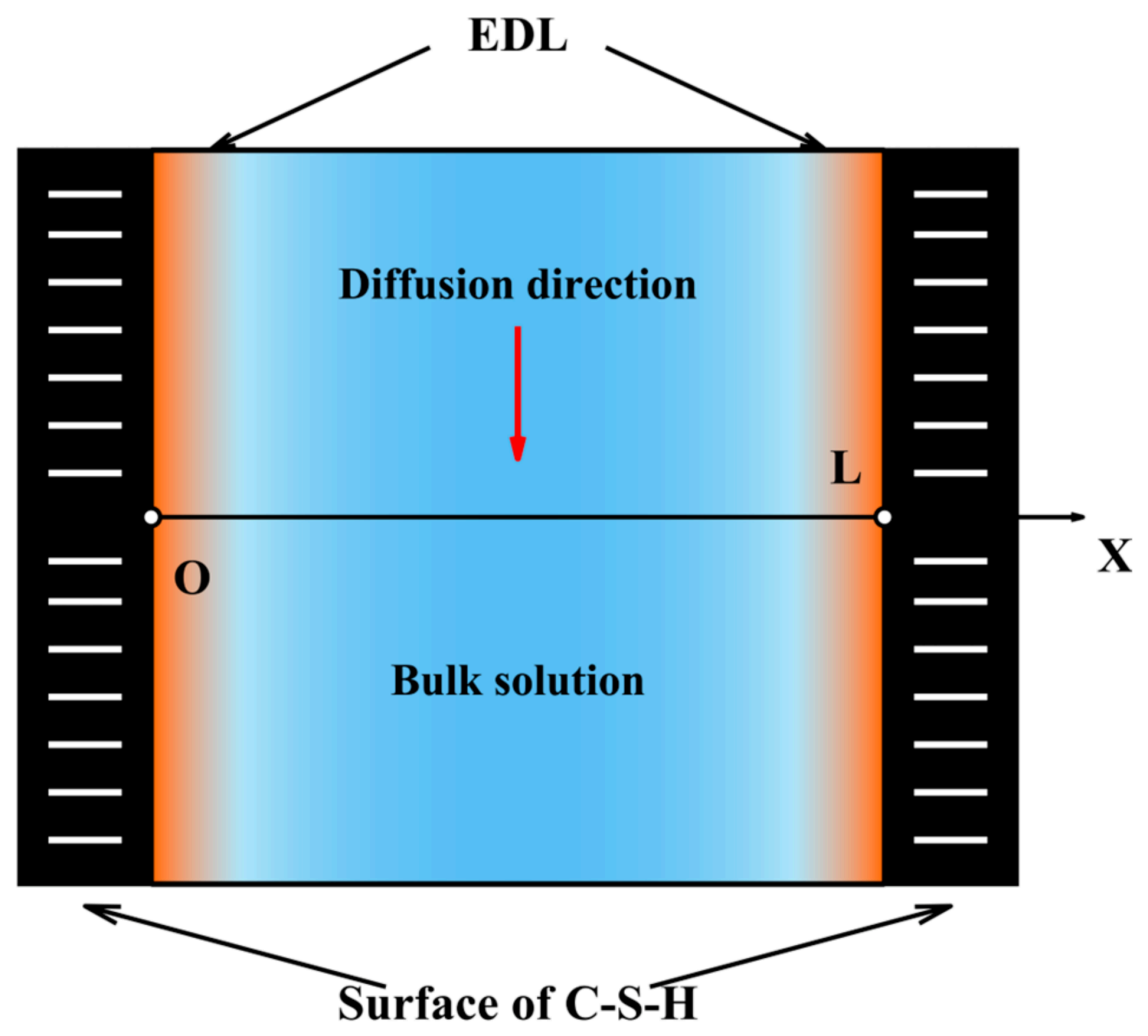
A 3D cylinder-shaped pore model of dynamic equilibrium diffusion with EDL phenomenon.

To evaluate the numerical accuracy and fidelity of Equation (7), a cylinder-shaped pore model was established based on the framework of Zhang et al. [9], as schematically depicted in Figure 7. Electrostatically, the diffuse-layer potential distribution is governed by the Poisson–Boltzmann (PB) model. Under typical cement pore solution conditions where the concentration of alkali metal cations (Na^+^, K^+^) is sufficiently high, the Stern layer surface potential is assigned a representative value of $\psi_{0}=-20\;\mathrm{mV}$ [9]. The Stern layer thickness is δ=0.8 nm and the Debye screening length is $\lambda_{D}=0.608\;\mathrm{nm}$ (corresponding to an ionic strength of I≈0.3–0.5 mol/L). To account for the finite size of hydrated ions, the classical point-charge PB solution is modified by incorporating the exact analytical steric hindrance polynomial derived by Dechadilok and Deen [50] for cylinder-shaped pores. It should be noted that this validation is performed for anionic species; the applicability of the simplified $\left|Z_{k}\right|$-dependent treatment to both cations and anions is further assessed at the cement paste scale (Table 3).

(8) $$H_{k}\left(\lambda \right)=\left(1-\lambda \right)\left(1-1.004\lambda +0.418\lambda^{3}+1.21\lambda^{4}-0.169\lambda^{5}\right)$$

where $\lambda =r_{k}/R_{c}$ is the ratio of the hydrated ion diameter to the pore width ($r_{k}$ = 0.25 nm, 0.38 nm, and 0.46 nm for mono-, di-, and trivalent ions, respectively). The total local relative diffusivity in the cylinder-shaped pore model is computed as the product of electrostatic confinement and hydrodynamic exclusion: $D_{\mathrm{local}}^{\mathrm{PB}+\mathrm{Steric}}\left(d,R_{c}\right)=H_{k}\left(\lambda \right)\cdot D_{local}^{PB}$.

**Figure 8:**
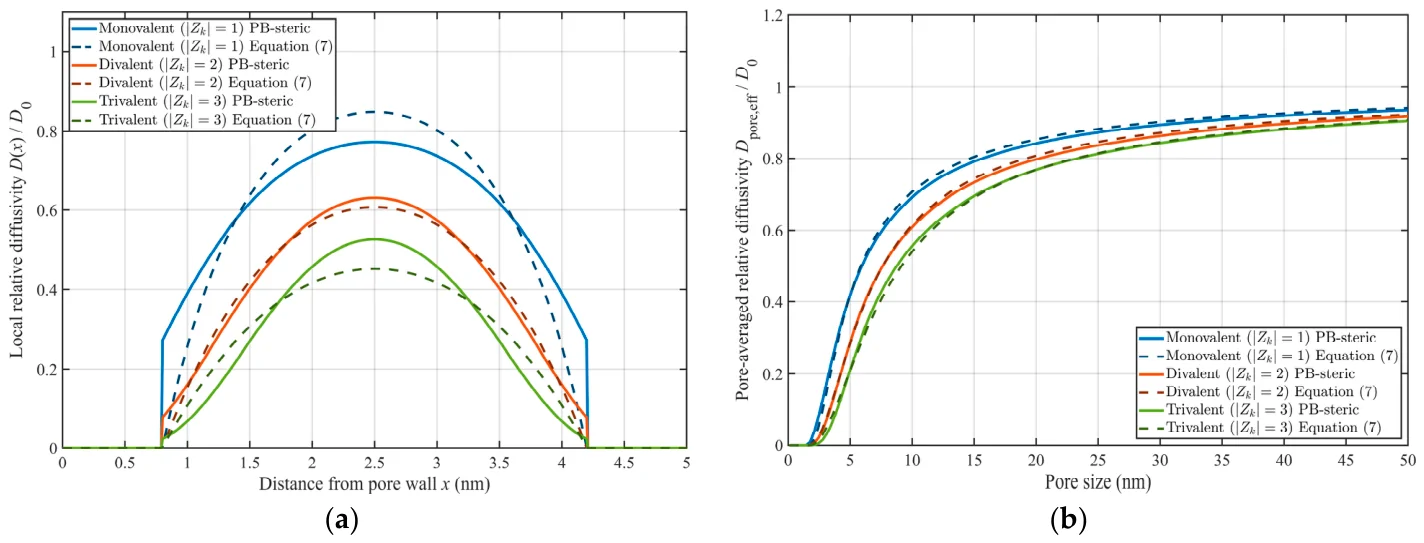
Quantitative validation of the analytical EDL model against the full PB-steric cylinder-shaped pore solution: (**a**) local relative diffusivity profile in a representative 5 nm cylinder-shaped pore; (**b**) pore-averaged effective relative diffusivity across the 1.5–50 nm pore size spectrum.

As depicted in Figure 8a for a representative 5 nm cylinder-shaped pore, the local diffusivity profile of the analytical model does not completely overlap with the cylinder-shaped pore solution due to inherent model simplifications. Near the solid boundary (d→δ), the analytical model enforces a strict zero-mobility boundary (D = 0), whereas the continuum PB model exhibits an artificial step-discontinuity. Although the local diffusion coefficients inside the pore show notable differences between the two models, both profiles demonstrate a consistent physical trend of increasing progressively from the solid wall toward the pore center. Furthermore, these localized profile differences are effectively smoothed out upon spatial integration. As shown in Figure 8b, the overall pore-averaged effective diffusivities for ions of all valences exhibit remarkable agreement with the pore across the entire 1.5–50 nm pore spectrum, yielding Mean Relative Errors (MRE) and Root Mean Square Errors (RMSE) of 2.95% (RMSE = 0.0106) for monovalent ($\left|Z_{k}\right|=1$), 1.93% (RMSE = 0.0079) for divalent ($\left|Z_{k}\right|=2$), and 6.01% (RMSE = 0.0077) for trivalent ions ($\left|Z_{k}\right|=3$), respectively. The slightly higher MRE for trivalent ions reflects their larger hydrated radii and stronger electrostatic interactions, which the analytical model simplifies through the sole parameter *κ* without resolving specific ion–surface correlations. Nevertheless, the MRE remains well within 10%, confirming the model’s adequacy for multi-scale reactive transport simulations where computational efficiency is paramount. This confirms that Equation (7) provides an optimal trade-off between physical fidelity and computational efficiency for multi-scale lattice calculations.

Overall, while this multi-scale calculation chain provides a computationally efficient scheme for evaluating ionic diffusivities, several inherent framework limitations should be recognized. At the lattice network level (Section 2.2.1), mass exchange is formulated on a 6-neighbor voxel grid using harmonic mean interfacial diffusivities, which simplifies off-axis anisotropic transport tensors and localized flow path tortuosity. At the nano-pore level (Section 2.2.2), the analytical EDL correction offers a practical first-order approximation based on wall distance and valence magnitude $\left|Z_{k}\right|$. However, it treats electrostatic confinement symmetrically regardless of charge sign, omitting specific ion–surface chemical adsorption polarities and dynamic hydration shell interactions. Furthermore, transferring properties across hierarchical levels via single effective diffusivities inevitably smooths out sub-scale microstructural heterogeneities. Acknowledging these simplifications clarifies the physical boundary of the multi-scale transport model while identifying targets for future micro-mechanical refinements.

## 3. Simulation Methods for Phase Evolution Under Multi-Ion Attack

After establishing the multi-scale calculation framework for ion diffusion coefficients, this section presents the transport theory for ions in a multi-ionic environment and, based on the thermodynamic equilibrium between liquid and solid phases, develops the methodology for simulating the phase evolution induced by chloride and sulfate ingress. A transport–reaction coupled model for cement paste subjected to multi-ion attack is thus constructed. At the macroscopic scale, a two-dimensional model of cement paste is built to analyse the degradation behaviour of cement paste under different water-to-cement ratios and exposure durations.

### 3.1. Governing Equations for Mass Transport

In a multi-ionic system, different ionic species possess distinct diffusion coefficients and charge numbers. When cations such as Na^+^ and Ca^2+^ migrate through the same pore channels as anions such as Cl^−^, OH^−^ and SO_4_^2−^, the significant differences in their migration rates (characterized by their diffusion coefficients) generate a charge imbalance that induces a local electrostatic potential gradient. The total flux of each ion must therefore account for both diffusion driven by the concentration gradient and electromigration driven by the electrostatic potential gradient. The Nernst–Planck equation provides the fundamental theoretical framework for describing mass transport of ions in a dilute solution under the combined influence of these two driving forces. For the i-th ionic species in the pore solution of cement paste, the total flux in the x direction (mol/(m^2^·s)) can be expressed as [49]

(9) $$J_{i}=-D_{i}\frac{\partial c_{i}}{\partial x}-\frac{z_{i}F}{RT}D_{i}c_{i}\frac{\partial \psi }{\partial x}$$

where $D_{i}$ is the effective diffusion coefficient of ion i in the pore solution of the cement paste (m^2^/s), $c_{i}$ is the concentration of ion i in the pore solution (mol/m^3^), $z_{i}$ is the charge number of ion i (dimensionless), F is the Faraday constant (96,485 C/mol), R is the ideal gas constant (8.314 J/(mol·K)), T is the thermodynamic temperature (298.15 K), and ψ is the local electrostatic potential (V).

The first term on the right-hand side of Equation (9) is the diffusion flux driven by the concentration gradient and obeys Fick’s first law. The second term is the electromigration flux driven by the electrostatic potential gradient. When ∂ψ/∂x≠0, cations ($z_{i}>0$) tend to migrate toward regions of lower potential, whereas anions ($z_{i}<0$) migrate toward regions of higher potential. The net effect is that both types of ions move in the same direction [51]. In the macroscopic transport simulation at the cement paste scale, the transport equations are based on the continuum hypothesis, i.e., the concentration of each ion at any point is the volume average over the REV at that location, and is therefore suitable for describing the spatial and temporal distributions of ions at the macroscopic scale. The EDL-induced spatial variation in the local diffusion coefficient near the pore wall is accounted for at the micro-scale through the analytical correction described in Section 2.2.2.

According to the principle of mass conservation, the rate of change in the concentration of ion i per unit volume of cement paste equals the divergence of its flux plus the source term due to chemical reactions,

(10) $$\frac{\partial \left(\varphi c_{i}\right)}{\partial t}=-\frac{\partial J_{i}}{\partial x}+\varphi G_{i}$$

where φ is the porosity of the cement paste (dimensionless) and $G_{i}$ is the chemical source term for ion i (mol/(m^3^·s)), representing the amount of ion i generated or consumed per unit time per unit volume of pore solution by chemical reactions. A positive value denotes net generation (e.g., Ca^2+^ released into the pore solution due to CH dissolution), and a negative value denotes net consumption (e.g., Cl^−^ consumed by the formation of Friedel’s salt).

Substituting Equation (9) into Equation (10) yields the coupled transport–reaction governing equation for ion i,

(11) $$D_{i}\frac{\partial^{2}c_{i}}{\partial x^{2}}+\frac{z_{i}F}{RT}D_{i}\frac{\partial }{\partial x}\left(c_{i}\frac{\partial \psi }{\partial x}\right)+\varphi G_{i}=\frac{\partial }{\partial t}\left(\varphi c_{i}\right)$$

In Equation (11), the spatial distribution of the electrostatic potential ψ remains unknown. To close the system of equations, the electroneutrality condition must be invoked. Since the pore solution of cement paste has a relatively high ionic strength, any charge imbalance is eliminated by ion migration on a time scale far shorter than the time step of ion transport [52]. The transient charge separation process can therefore be neglected, and the local electroneutrality constraint is imposed directly,

(12) $$\sum_{i}z_{i}c_{i}=0$$

Equation (12) implies that the net charge density at any spatial location in the cement paste is zero. Taking the time derivative of this constraint and substituting Equation (12) yields, after algebraic manipulation, an explicit expression for the electrostatic potential gradient [51],

(13) $$\frac{\partial \psi }{\partial x}=\frac{RT}{F}\cdot \frac{\sum_{i}z_{i}D_{i}\frac{\partial c_{i}}{\partial x}}{\sum_{i}z_{i}^{2}D_{i}c_{i}}$$

Substituting Equation (13) back into Equation (9) allows the total flux of each ion to be computed directly under the combined effects of diffusion and electromigration, without explicitly solving the Poisson equation. For the multi-ionic system studied in this work, the complete set of governing equations is formed by coupling the partial differential equations for all relevant ions (Equation (11)) with the electroneutrality condition (Equation (13)), and this system describes the multi-ion coupled transport in the pore solution.

### 3.2. Thermodynamic Equilibrium Reaction System

Under conditions of constant temperature (25 °C) and pressure, the equilibrium state of any chemical reaction in a cementitious system is described by the law of mass action. For a generic reaction of the form

(14) $$\sum_{i}v_{i}A_{i}=0$$

where $A_{i}$ denotes the chemical species participating in the reaction (including solid phases and aqueous ions) and $v_{i}$ is the stoichiometric coefficient (negative for reactants, positive for products), the reaction quotient must equal the equilibrium constant at thermodynamic equilibrium [26,53],

(15) $$K_{p}=\prod_{i}{\left(\gamma_{i}c_{i}\right)}^{v_{i}}$$

where $c_{i}$ is the concentration of species i (mol/L) and $\gamma_{i}$ is its activity coefficient. For solutions with high ionic strength such as cement pore solutions (0.3–0.5 mol/L), the activity coefficient is often calculated using the Davies equation or the extended Debye–Hückel equation. When the reaction quotient is lower than $K_{p}$, the reaction proceeds in the forward direction (dissolution of the solid phase); when it exceeds $K_{p}$, the reaction proceeds in the reverse direction (precipitation of the solid phase).

In a multi-ionic system, the individual reactions are not independent; multiple chemical reactions share the same pore solution ions and solid phases. For instance, the Ca^2+^ concentration in the pore solution simultaneously affects the dissolution equilibrium of CH, the decalcification equilibrium of C-S-H, and the formation equilibrium of gypsum. The thermodynamic state of the entire system must therefore be solved by simultaneously satisfying the mass action law for all reactions and the mass conservation for all elements [32]. Table 4 lists the principal chemical reaction systems involved in chloride and sulfate attack and their thermodynamic equilibrium constants, as adopted in the present model. The model also includes physical binding effects of other ions as part of the overall phase equilibrium calculation.

### 3.3. Influence of Aggressive Ions on Multi-Scale Phase Evolution

#### 3.3.1. Chloride Ingress and Friedel’s Salt Precipitation

When chloride ions penetrate the cement paste, they react with AFm to form Friedel’s salt (3CaO·Al_2_O_3_·CaCl_2_·10H_2_O) [13]. This is an in situ solid-phase transformation in which Cl^−^ displaces SO_4_^2−^ from the interlayer of AFm, yielding the chemically more stable Friedel’s salt. The molar volume of Friedel’s salt (approximately 350 cm^3^/mol) is about 1.12 times that of AFm (approximately 313 cm^3^/mol), corresponding to a solid volume expansion of approximately 12%.

The formation of Friedel’s salt is directly linked to the presence of AFm, which is predominantly formed in the outer hydration product layer during the middle and later stages of cement hydration. It should be acknowledged that the present multi-scale microstructure model does not resolve individual mineral phases such as AFm. Instead, all hydration crystalline phases are collectively represented as a single phase distributed around the capillary pores at both the cement paste scale (Level 3) and the outer C-S-H scale (Level 2). Since AFm, the dominant reactant for Friedel’s salt formation, is primarily located in these regions, this simplification is considered reasonable. The crystal growth algorithm is therefore constructed as follows. At Level 3, voxels of the hydration crystalline phase face-adjacent to large capillary pores are randomly selected as nucleation sites, and Friedel’s salt expands outward, compressing the surrounding pore space. At Level 2, Friedel’s salt grows from the LD C-S-H region into the small capillary pores, locally filling the pore channels. Newly formed Friedel’s salt preferentially attaches to voxels already occupied by Friedel’s salt, consistent with the general principle of crystal growth from existing nuclei. During dissolution, the reverse sequence is applied.

Once the quantity of Friedel’s salt to be precipitated is determined from the thermodynamic equilibrium calculation, a dynamic allocation scheme is employed to distribute it between Level 3 and Level 2, based on the solid–liquid contact areas at the two scales. Let $A_{level3}$ and $A_{level2}$ denote the contact areas of large and small capillary pores, respectively. The allocation coefficient for Level 3 is

(16) $$\eta_{level3}=\frac{A_{level3}}{A_{level3}+A_{level2}}$$

and that for Level 2 is $\eta_{level2}=1-\eta_{level3}$. If the allocated volume exceeds the available pore space at either scale, the excess is redirected to the other scale. This dual-scale allocation with overflow redirection ensures mass conservation while respecting the physical capacity limits of the pore spaces.

Owing to the limited volumetric expansion of only 12% and the relatively low AFm content in hydrated cement paste, the pore-filling effect of Friedel’s salt is inherently moderate. The filling occurs primarily in the small capillary pores of the outer C-S-H layer, with minimal impact on the percolation of the large capillary pore network.

**Figure 9:**
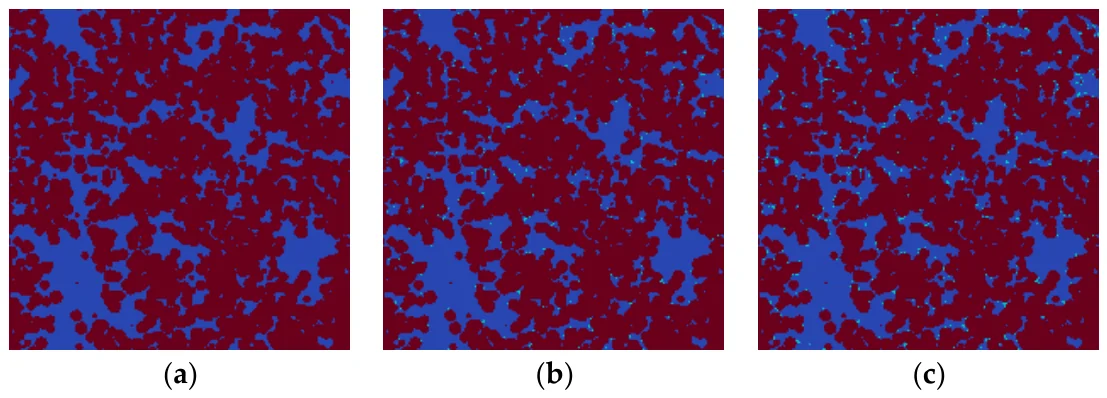
Evolution of the Level 2 two-dimensional slices during Friedel’s salt deposition: (**a**) $∆V_{FS}$ = 0 mol/m^3^; (**b**) $∆V_{FS}$ = 75 mol/m^3^; (**c**) $∆V_{FS}$ = 150 mol/m^3^.

Figure 9 illustrates the microstructural evolution during Friedel’s salt deposition at Level 2 (w/c=0.4). Newly formed Friedel’s salt is shown in light blue, small capillary pores in dark blue, and LD C-S-H in reddish brown. At the early stage, Friedel’s salt precipitates as discrete light blue voxels near the original AFm sites, partially filling some of the dark blue small capillary pore channels. As deposition progresses, the light blue Friedel’s salt clusters grow larger and additional nucleation sites appear, further encroaching upon the dark blue pore space. Nevertheless, the overall percolation of the capillary pore network is not substantially affected.

#### 3.3.2. Sulfate Attack and Ettringite Formation

The core mechanism of sulfate attack is the reaction between SO_4_^2−^ and AFm to form ettringite (AFt), with the solid molar volume expanding from approximately 313 cm^3^/mol to 735 cm^3^/mol, a volumetric expansion of 135% [16,17]. Microscopic observations confirm that ettringite preferentially precipitates in capillary pores and the surrounding outer hydration products, significantly altering the capillary porosity [16].

The evolution of sulfate attack exhibits two distinct stages. In the first stage, ettringite fills capillary pores without generating significant stress, causing densification. In the second stage, continued crystal growth in the confined pore space generates crystallisation pressure exceeding the local tensile strength, and microcracks nucleate. These cracks originate from ettringite-filled pores and propagate into the surrounding hydration products, rarely penetrating unhydrated cement cores [16].

The crystal precipitation algorithm follows the same logic as that for Friedel’s salt (Section 3.3.1), and the allocation between Level 3 and Level 2 follows Equation (16). Physically, ettringite initially precipitates within capillary pores without inducing immediate expansion, providing porosity-dependent buffering capacity against crystallization pressure. Porosity evolution data and combined chloride–sulfate exposure tests [54,55] show that microcracking initiates when the cumulative capillary pore filling fraction reaches ~40%. This mechanism is supported by the chemo-mechanical model of Tixier and Mobasher [56,57], where the pre-expansion pore filling fraction f ranges from 0.35 to 0.55. Sensitivity analysis [57] confirms that a larger f provides more buffering space, delaying stress accumulation and lowering both crack generation rates and final crack volumes, whereas a smaller f induces earlier microcracking. Based on these experimental and theoretical insights, $V_{\mathrm{crit}}$ is set at 40% of the initial capillary pore volume ($\varphi_{\mathrm{cap}}$).

The microcrack geometry is defined using fracture mechanics and microstructural constraints. Following the crack band model ($G_{f}=g_{t}\cdot w$) of Gu et al. [58], the characteristic crack width is set to w = 2 μm to ensure energy dissipation equivalence. Regarding microcrack length, crack propagation is primarily governed by pore network topology, solid phase distribution, localized stress fields, and matrix fracture properties, with modeling approaches varying across characteristic scales. Within typical micro-scale RVE domains (100–200 μm) [59,60], forming a percolating transport path bridging adjacent matrix interfaces requires a crack trajectory spanning several tens of micrometers (50–100 μm). Thus, w=2  μm and L=60 μm are selected as representative parameters for a typical microcrack.

When ettringite volume exceeds $V_{\mathrm{crit}}$, excess volume drives microcracking via $V_{\mathrm{crack}}=\beta \cdot \left(V_{\mathrm{ettringite}}-V_{\mathrm{crit}}\right)$. Based on Gu et al. [58], the ratio of crack volume to internal crystal volume equals $1/S_{C}$, where crystal saturation $S_{C}$ (i.e., the fraction of the pore volume that can be filled by crystals before further growth exerts pressure) ranges from 0.05 to 0.45 during ingress. Adopting the median $S_{C}=0.25$ yields $\beta =1/S_{C}=4.0$, which aligns with cross-disciplinary inclusion expansion models [61,62,63] where crack-tip stress concentrations cause crack void growth to exceed stoichiometric product growth. Qualitatively, a higher β accelerates post-threshold crack volume accumulation and steepens diffusivity deterioration, whereas a lower β moderates crack volume growth and yields a smoother transition. Using a constant median β=4.0 avoids tracking dynamic $S_{C}$ evolution, offering a computationally efficient macroscopic approximation.

Nevertheless, the formulation of prescribed crack geometry and a constant β represents a macroscopic equivalent simplification. The present model is strictly confined to paste-scale RVE representations, whereas actual engineering concrete structures exhibit cracking phenomena spanning multiple spatial orders of magnitude, from micro-scale matrix damage to macro-scale structural fractures. Furthermore, adopting a static median β cannot resolve the time-dependent variation in crack generation rates during exposure. For finer resolution of spatial crack tortuosity, branching, and multi-scale damage, advanced discrete crack models [44] or phase-field fracture approaches can be incorporated in future work. For the macroscopic diffusion prediction intended here, these simplifications capture the first-order effect of cracking on diffusivity. More refined crack representations would primarily improve the local damage pattern rather than the overall transport trend.

**Figure 10:**
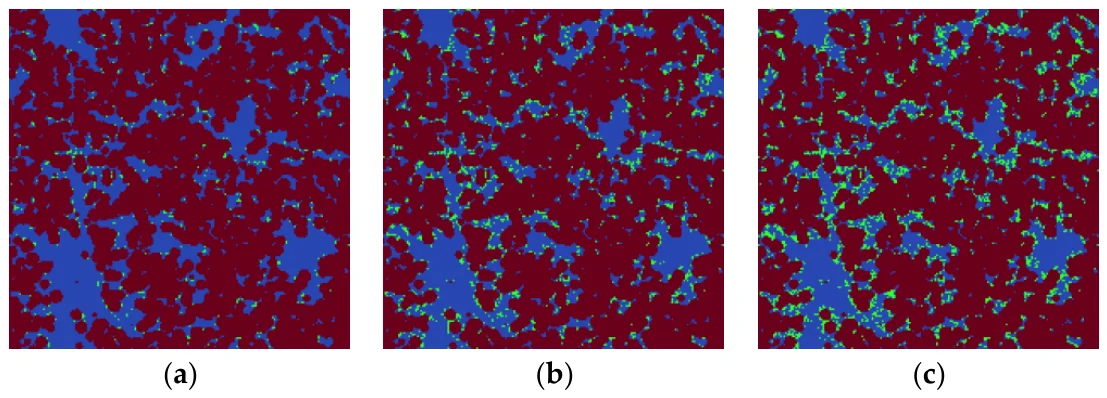
Evolution of the Level 2 two-dimensional slices during ettringite deposition: (**a**) $∆V_{\mathrm{Ettr}}=$ 20 mol/m^3^; (**b**) $∆V_{\mathrm{Ettr}}$ = 60 mol/m^3^; (**c**) $∆V_{\mathrm{Ettr}}$ = 100 mol/m^3^.

Figure 10 illustrates ettringite deposition at Level 2 (w/c=0.4), with newly formed ettringite in green. The precipitation pattern resembles that of Friedel’s salt, but the 135% expansion causes a far larger impact on the pore space. At high ettringite contents, substantial precipitation blocks portions of the small capillary pores.

**Figure 11:**
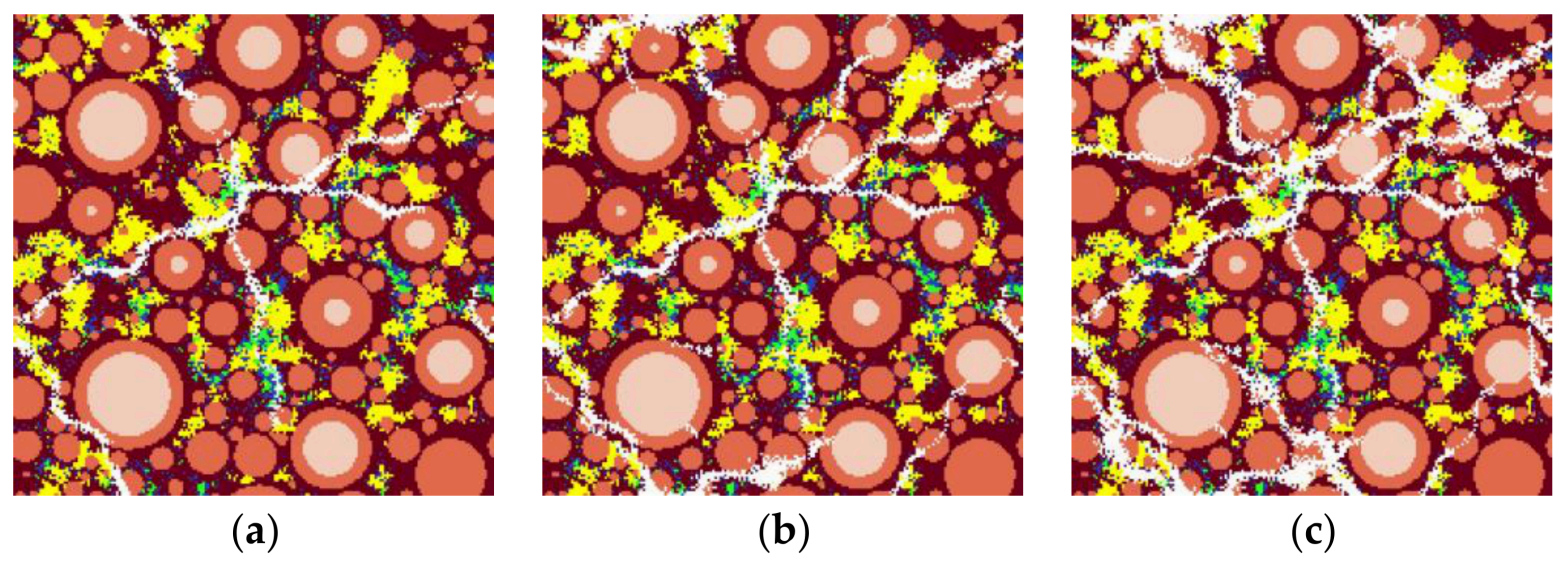
Evolution of the Level 3 two-dimensional slices of crack development during ettringite deposition: (**a**) $∆V_{\mathrm{Ettr}}=$ 140 mol/m^3^; (**b**) $∆V_{\mathrm{Ettr}}$ = 180 mol/m^3^; (**c**) $∆V_{\mathrm{Ettr}}$ = 220 mol/m^3^.

Figure 11 illustrates crack network evolution at Level 3 (w/c=0.4) once $V_{\mathrm{crit}}$ is exceeded, where microcracks generated at the cement paste scale are shown in white. Cracks initiate in ettringite-rich regions and gradually interconnect along filled pore zones, while unhydrated cores remain intact. The effect on the diffusion coefficient is non-monotonic: pore filling reduces it in the early stage, and the crack network sharply increases it in the later stage.

#### 3.3.3. Calcium Leaching

Calcium leaching is the process in which Ca^2+^ in the pore solution diffuses outward under a low external concentration, and calcium-bearing solid phases dissolve successively under thermodynamic disequilibrium [21,22,23]. The model simulates the complete leaching process from the dissolution of hydration crystalline phases to the full decalcification of C-S-H.

A random dissolution algorithm [35] is adopted. A voxel representing a dissolvable phase adjacent to a pore voxel is eligible for dissolution, and the required number of voxels is randomly selected from all eligible sites. The amount to be dissolved is determined from the thermodynamic equilibrium calculations described in Section 3.2. The dissolution proceeds in a sequential manner: hydration crystalline phases at Level 3 dissolve first, followed by those at Level 2. CH crystals dissolve preferentially from voxels exposed to pores. For C-S-H, LD C-S-H dissolves before HD C-S-H, reflecting the higher chemical stability of the latter.

**Figure 12:**
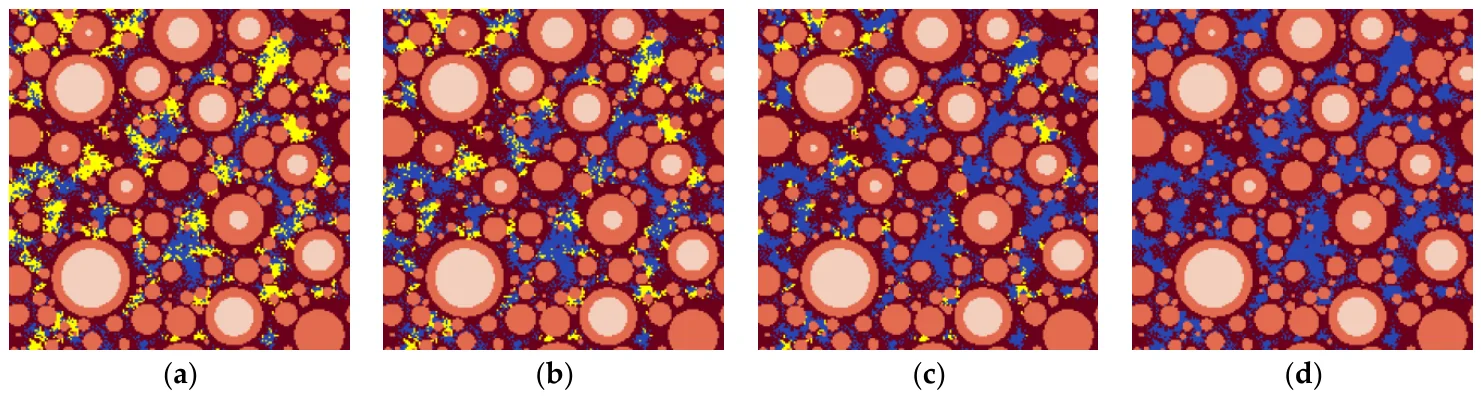
Evolution of the Level 3 two-dimensional slices during calcium leaching of the hydrated crystalline phases: (**a**) $∆V_{HC}=-25\%V_{HC}$; (**b**) $∆V_{HC}=-50\%V_{HC}$; (**c**) $∆V_{HC}=-75\%V_{HC}$; (**d**) $∆V_{HC}=-100\%V_{HC}$.

Under the conditions considered in this study, the impact of calcium leaching is primarily manifested as the progressive dissolution of CH at Level 3. Figure 12 illustrates this process at Level 3 (w/c=0.4) at increasing leaching levels. As leaching progresses, CH crystals are gradually dissolved, with surface-exposed voxels preferentially removed. As the hydration crystalline phases approach complete dissolution, the volume of large capillary pores increases noticeably and pore connectivity improves. These changes directly increase the effective diffusion coefficient. C-S-H decalcification, occurring at more advanced stages, further coarsens the gel pore structure and contributes to an additional increase in diffusivity.

### 3.4. Macroscopic Diffusion Model and Validation

**Figure 13:**
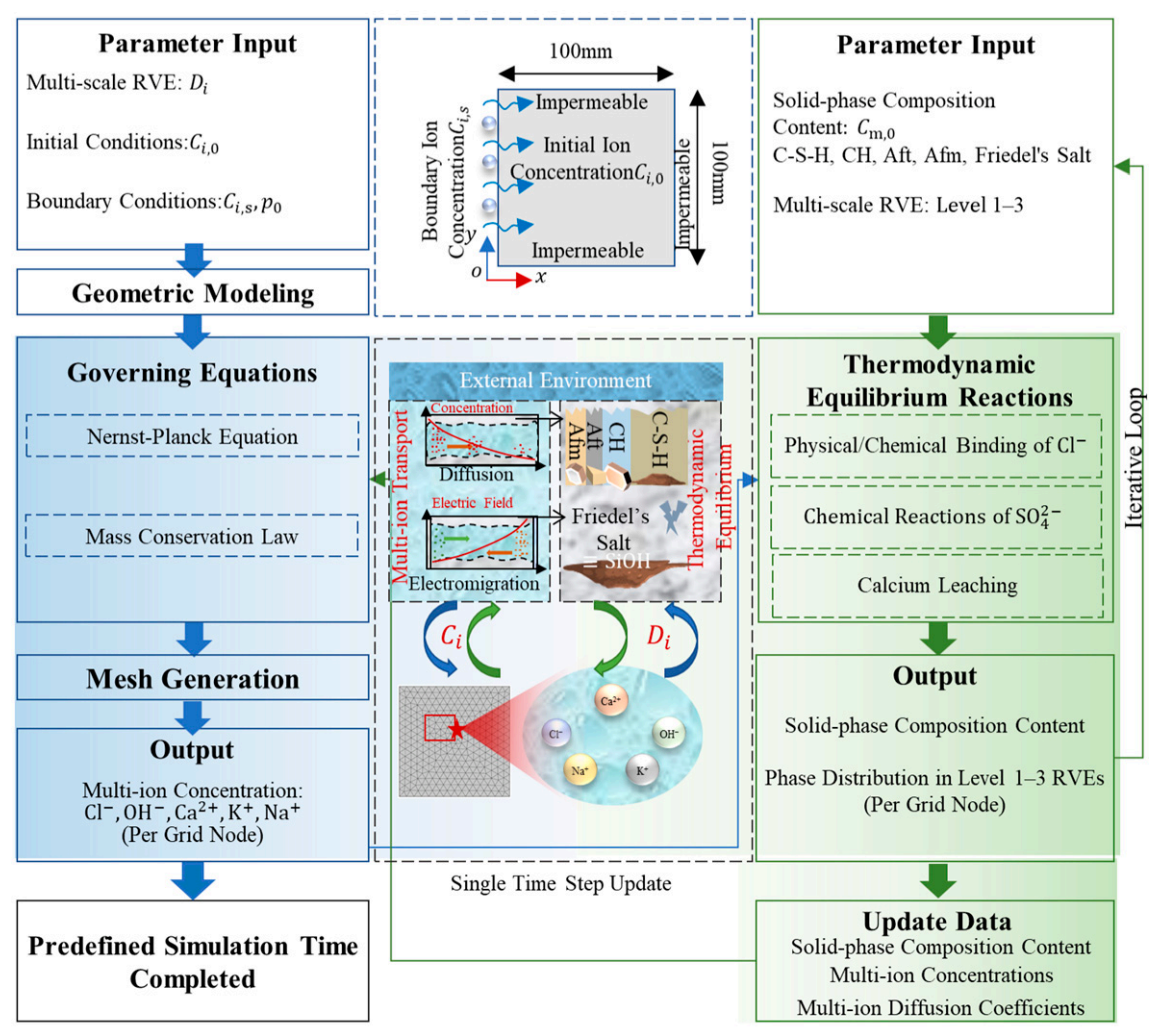
Modelling of multi-ion transport with transport-reaction coupling.

A multi-ion transport–reaction coupled model was established for the simultaneous transport of chloride and sulfate ions in cement paste, with full consideration of the intrinsic coupling between multi-ion transport and physicochemical reactions. The operational logic of the macroscopic diffusion model is illustrated in Figure 13. The model consists of two modules: a multi-ion transport module and a thermodynamic equilibrium reaction module. In each iteration, the transport module updates the spatial distribution of ion concentrations over the current time step. The updated concentrations are then passed to the thermodynamic equilibrium module, where the solid phase composition and ion concentrations at each grid point are adjusted according to the reaction calculations. The phase distributions in the multi-scale RVEs are updated accordingly, and the diffusion coefficients are recalculated. These updated coefficients and ion concentrations are then fed back to the transport module for the next iteration. In this manner, the transport and reaction modules are coupled through the multi-ion concentration field and the multi-scale microstructural state.

The reaction module encompasses not only the three-phase evolution processes explicitly modelled at the RVE scale in Section 3.3 (Friedel’s salt precipitation, ettringite formation, and calcium leaching), but also the formation and dissolution of other relevant phases, including Kuzel’s salt, gypsum, and additional hydration products. The phase evolution algorithms for these additional phases follow the same modelling principles as those described in Section 3.3: the amount of each phase to be formed or dissolved is determined from the thermodynamic equilibrium calculations, and the associated volume changes are allocated between Level 3 and Level 2 according to Equation (16). This unified treatment ensures that the multi-scale RVE framework consistently captures the porosity evolution driven by all chemical reactions involved.

Within each reaction time step, the sequence of phase transformations follows a prescribed order: dissolution reactions are executed before precipitation reactions. This sequence reflects the physical reality that dissolution of existing solid phases, which supplies ions to the pore solution, must precede the formation of new phases that consume those ions. Among dissolution reactions, the order is determined by thermodynamic stability: phases with higher solubility dissolve before those with lower solubility, as the former depart from equilibrium first when the pore solution composition shifts. Among precipitation reactions, the phase whose saturation index is exceeded by the largest margin precipitates first. It should be acknowledged that this sequential treatment represents a simplification of the true coupled dissolution–precipitation process, which occurs simultaneously under a global Gibbs free energy minimisation constraint. However, given that the reaction time step is much shorter than the characteristic time of ion transport, and that the local chemical environment evolves gradually, the sequential approximation is considered acceptable for the present purpose.

A two-dimensional macroscopic model measuring 100 mm × 100 mm was established for the demonstration of the coupled framework. Ion ingress is permitted through one face, and the remaining three faces are set as impermeable. The input parameters include material properties, environmental conditions, initial conditions, and boundary conditions. The initial multi-ion diffusion coefficients were obtained from the multi-scale calculations described in Section 2.2. The initial solid phase contents were extracted from the Level 1–3 RVEs based on the cement hydration chemistry. Following the typical pore solution composition of hydrated cement paste and considering similar marine environments and material properties, the initial pore solution concentrations were set as $C_{\mathrm{Cl},0}=0$ mol/m^3^, $C_{\mathrm{Na},0}=35$ mol/m^3^, $C_{\mathrm{OH},0}=78$ mol/m^3^, $C_{K,0}=35$ mol/m^3^, and $C_{\mathrm{Ca},0}=4$ mol/m^3^, with reference to existing literature. To represent the combined ingress of sodium, chloride, and sulfate ions, the boundary concentrations were set as $C_{\mathrm{Cl},s}=845$ mol/m^3^, $C_{\mathrm{SO},s}=31$ mol/m^3^, and $C_{\mathrm{Na},s}=907$ mol/m^3^, with all other ion boundary concentrations set to 0 mol/m^3^.

Given the large number of grid points in the macroscopic model, performing a full RVE simulation for phase evolution and diffusion coefficient calculation at every grid point after each iteration would be computationally prohibitive. A phase evolution database was therefore constructed to enable efficient retrieval of the diffusion coefficient evolution. Prior to the coupled simulation, RVEs covering the typical reaction extents of all relevant ions were pre-computed. During the simulation, the relative diffusion coefficient is retrieved from the database by interpolation, using the volume change in each solid phase $\Delta V_{j}$ as the independent variable, where the subscript j denotes different solid phase species. A predefined tolerance is set; when the deviation between the current $\Delta V_{j}$ and the nearest entry in the database exceeds this tolerance, a new RVE simulation is triggered for the current phase composition and diffusion coefficient, and the result is appended to the database. This on-demand database expansion strategy significantly accelerates the computation while maintaining acceptable accuracy. To quantitatively evaluate the computational efficiency of the database strategy, a performance test was conducted against the full RVE recalculation scheme (recalculating multi-scale microstructures and lattice networks at every grid node per step). For a 100-day multi-ion attack simulation on a 100 mm × 100 mm domain, performing full RVE recalculations requires ~42.6 h of CPU execution time, whereas the database strategy reduces this to ~2.1 h on a workstation (Intel Core i9-13900K CPU @ 3.0 GHz, 64 GB RAM). This achieves a ~20-fold computational speedup and saves over 95% of CPU time while maintaining high precision.

While the coupled transport–reaction framework and database strategy enable dynamic calculation of diffusion coefficients and phase evolution during the transport process, certain system-level simplifications must be noted. Macroscopically (Section 3.1), ionic transport is governed by the continuum Nernst–Planck formulation with an instantaneous electroneutrality constraint, which neglects transient local charge separation dynamics occurring on sub-grid time scales. Chemically (Section 3.2 and Section 3.4), the framework assumes local thermodynamic equilibrium and prescribed sequential dissolution–precipitation reaction steps under isothermal conditions (${25\;}^{{}^{\circ}}C$), thereby omitting non-equilibrium reaction kinetics and field temperature fluctuations. Microstructurally (Section 3.3), the dynamic allocation of product volume and database-driven parameter interpolation simplify complex 3D crystal growth tortuosity, spatial branching, and leaching-induced skeleton hollow-out into macroscopic equivalent state variables. Recognizing these operational boundaries is essential for properly interpreting multi-ion attack simulations and guiding future multi-physics extensions.

**Figure 14:**
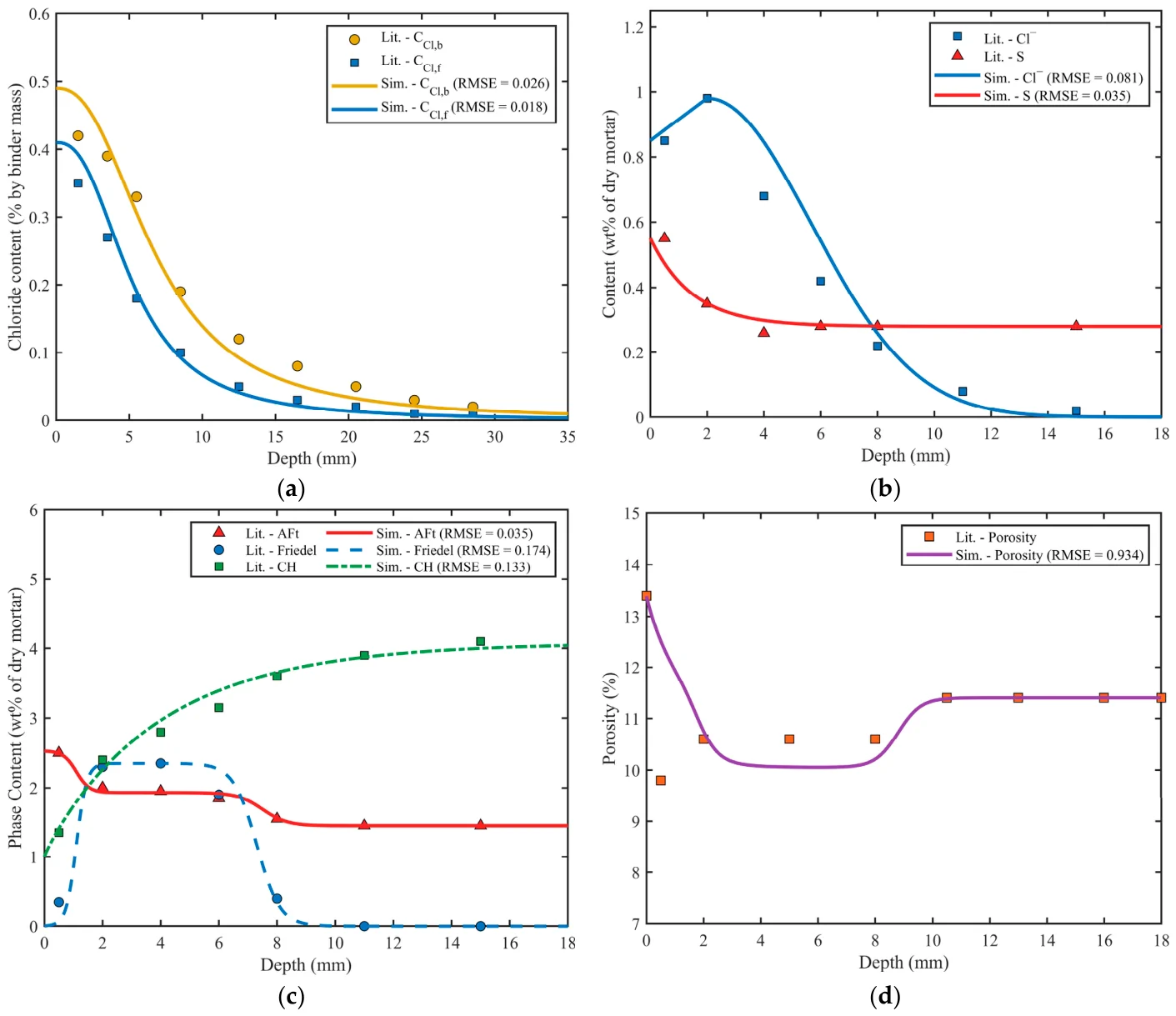
Experimental validation of the proposed model under various exposure conditions: (**a**) Bound and free chloride concentration profiles under pure chloride exposure [64] (72 d); (**b**) Total chloride and total sulfur content profiles under simulated seawater exposure [11] (180 d); (**c**) phase evolution profiles under simulated seawater exposure [11] (90 d); (**d**) Microstructural porosity evolution profile under combined chloride–sulfate exposure [49] (300 d).

To evaluate the predictive accuracy of the proposed model, numerical results were systematically compared with benchmark experimental datasets from the literature. During model setup, the initial cementitious phase compositions were calibrated to account for variations in clinker formulations and hydration degrees among different studies. For mortar specimens, a composite homogenization scheme was applied to consider the tortuosity hindrance and dilution effects of aggregates on ion diffusion. Under pure chloride exposure for 72 d (as shown in Figure 14a [64]), the model accurately reproduced the surface binding plateau and inward attenuation of both free and bound chlorides; the precise distinction and fitting of these distinct chloride forms convincingly validate the thermodynamic phase-equilibrium-based chloride binding formulation.

Under simulated seawater/combined chloride–sulfate exposure for 180 d (as shown in Figure 14b [11]), the model successfully predicted the subsurface chloride concentration peak, which primarily arises from the competitive displacement of bound chloride by sulfate ions reacting with AFm near the exposed surface. Meanwhile, the model accurately captured the total sulfur (S) profile, depicting its surface accumulation (0–4 mm) due to sulfate reaction product formation and its subsequent gradual decay to the sound matrix baseline (0.28 wt%). Under combined exposure for 90 d (as shown in Figure 14c [11]), the model achieved satisfactory fitting for the leaching-induced CH profile increasing from the surface inward, as well as the characteristic “double-step” spatial zonation of ettringite (AFt). For Friedel’s salt, the model captured its subsurface plateau profile, which forms via rapid reaction of penetrating chlorides and depletes near the surface due to sulfate substitution forming AFt. Note that experimental AFt and Friedel’s salt contents in the literature were extracted via thermal gravimetric analysis, where overlapping dehydration temperature ranges induce minor analytical deviations.

Regarding microstructural evolution under 10%${\mathrm{Cl}}^{-}$ + 5%${\mathrm{SO}}_{4}^{2-}$ exposure for 300 d (as shown in Figure 14d [49]), the model successfully captured the localized porosity variation near the outermost surface caused by calcium leaching, as well as the significant pore-filling effect in the subsurface region induced by the precipitation of AFt and Friedel’s salt (yielding a distinct porosity minimum plateau). Small discrepancies between simulated and experimental profiles are attributed to the simplified homogenization treatment of aggregate spatial distribution in the mortar composite model, and slight deviations between the local thermodynamic equilibrium assumption and actual chemical reaction kinetics. Overall, judging from the RMSE and high coefficients of determination for concentration and mineral phase profiles (R^2^ > 0.87, reaching up to 0.98), the proposed model demonstrates strong quantitative accuracy and robust physical self-consistency.

## 4. Results and Discussion

### 4.1. Free Ion Concentration Distributions in the Pore Solution

**Figure 15:**
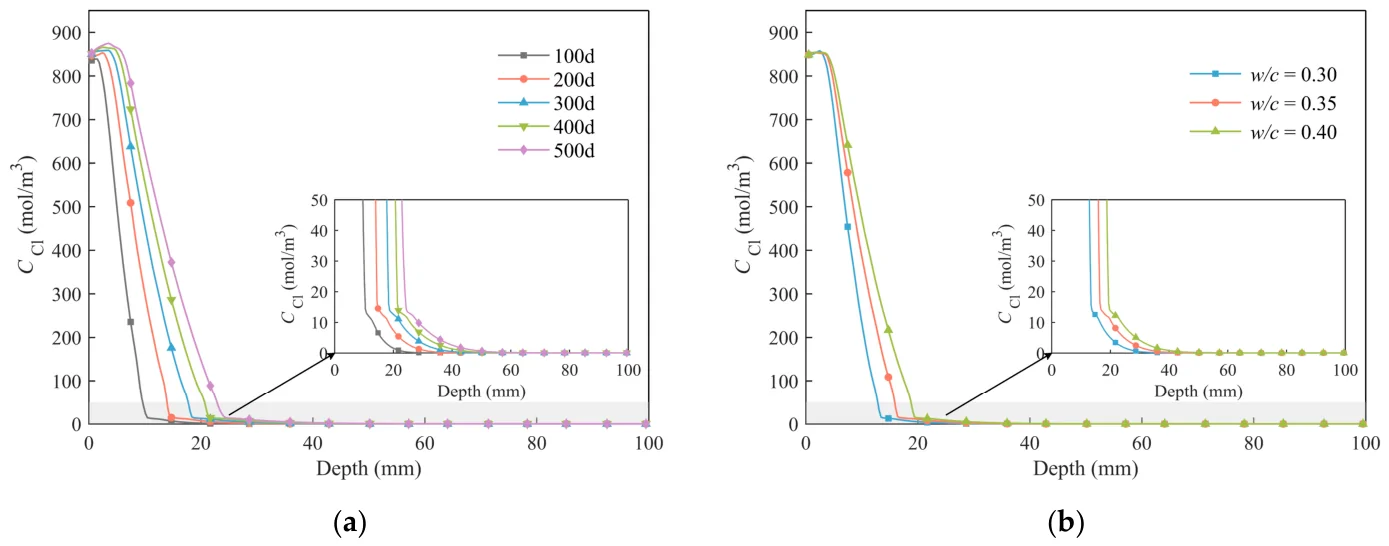
Variation in free chloride ion concentration with water-to-cement ratio and exposure time: (**a**) w/c=0.35; (**b**) 250 d.

Figure 15 presents the distribution characteristics of free chloride ions within the cement paste under different exposure durations and water-to-cement ratios. The concentration profiles exhibit three distinct zones: a high-concentration surface zone, a steep-gradient intermediate zone, and a low-concentration tail in the deeper region. The surface peak arises from the displacement effect of sulfate ingress, the steep intermediate gradient results from rapid chloride binding with AFm to form Friedel’s salt driven by the concentration gradient, and the low deep-region concentration is maintained by chemical and physical binding. As the exposure duration increases (Figure 15a), the concentration front advances continuously inward, but the rate of advance gradually slows owing to pore blockage by reaction products. A lower water-to-cement ratio (Figure 15b) produces a steeper intermediate gradient and a reduced penetration depth, primarily because the denser pore structure and greater amount of binding phases increase the resistance to transport and the efficiency of chloride binding.

**Figure 16:**
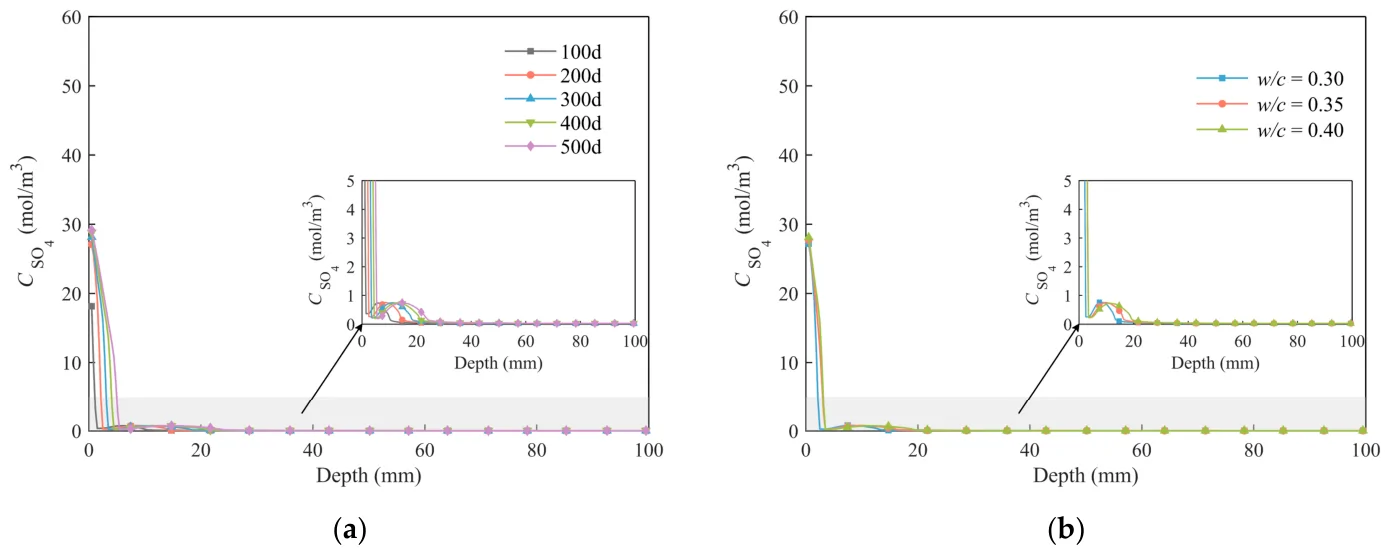
Variation in free sulfate ion concentration with water-to-cement ratio and exposure time: (**a**) w/c=0.35; (**b**) 250 d.

Figure 16 presents the distribution of free sulfate ions under different exposure durations and water-to-cement ratios. In the near-surface region (0–5 mm), the sulfate concentration decreases sharply, mainly because the intruding sulfate is rapidly consumed by the formation of ettringite. In the deeper region (10–30 mm), a distinct secondary enrichment peak can be observed in the magnified view, originating from chloride ions displacing bound sulfate from AFm and releasing it into the pore solution. As the exposure duration increases (Figure 16a), this displacement peak advances continuously inward, and its migration trajectory closely follows the chloride penetration front. A higher water-to-cement ratio (Figure 16b) increases both the depth and the magnitude of this secondary peak, reflecting the fact that lower transport resistance and better connectivity facilitate the migration and accumulation of the displaced sulfate in the pore solution.

**Figure 17:**
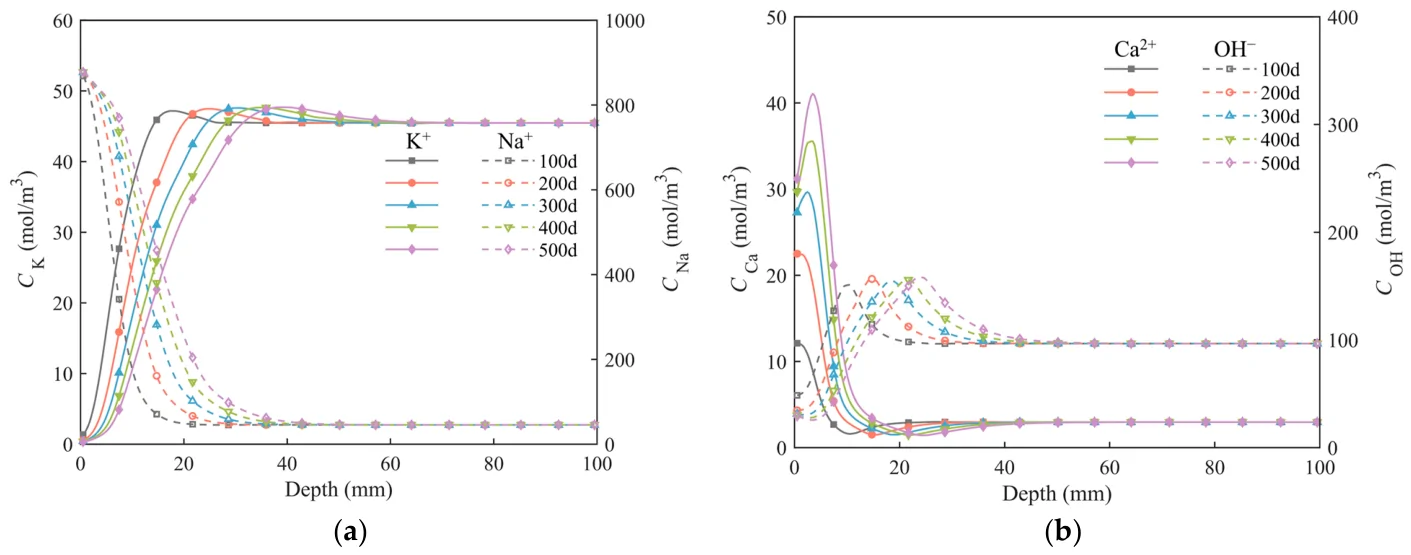
Variation in the concentrations of four major ions with exposure time (w/c = 0.35): (**a**) ${\mathrm{Na}}^{+},\;K^{+}$; (**b**) ${\mathrm{Ca}}^{2+},{\mathrm{OH}}^{-}$.

Figure 17 illustrates the coupled transport characteristics of Na^+^, Ca^2+^, OH^−^, and K^+^ within the cement paste. In the surface region (0–10 mm), the ingress of high-concentration Na^+^ increases the ionic strength of the pore solution, while the outward diffusion of OH^−^ lowers the pH. Together, these effects accelerate the dissolution of CH and produce a distinct surface enrichment peak of free Ca^2+^ (Figure 17b). At depths of 15–25 mm, the Ca^2+^ concentration drops sharply to a trough, mainly because the displaced sulfate reacts with Ca^2+^ in the pore solution to form ettringite, consuming a large amount of free calcium. In addition, electrochemical coupling significantly modulates the migration behaviour of the ions. The rapidly diffusing high-concentration Na^+^ (Figure 17a) generates an electrostatic drag that pulls the highly mobile OH^−^ inward, forming an enrichment peak, while the resulting potential gradient repels the intrinsic K^+^ toward the deeper region. These observations indicate that ion transport in a multi-ionic system is profoundly governed by the combined effects of phase transformations and electroneutrality constraints.

**Figure 18:**
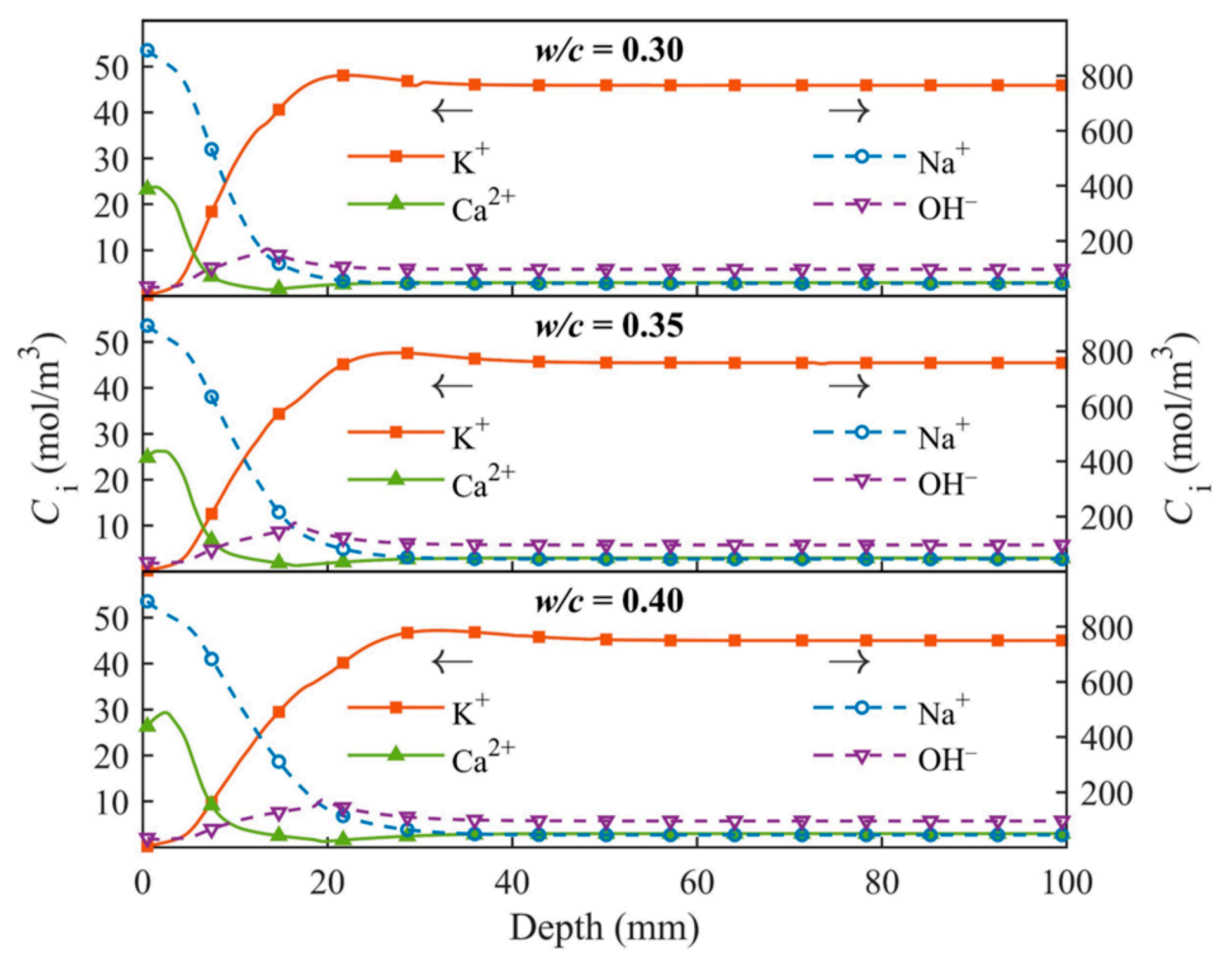
Variation in four ion concentrations *C*_i_ with cement paste depth under the influence of different water-to-cement ratios (250 d).

Figure 18 presents the concentration distributions of the four major ions at an exposure duration of 250 days for three water-to-cement ratios (0.30, 0.35, and 0.40). As the water-to-cement ratio increases, the capillary porosity and pore connectivity of the matrix increase, reducing the transport resistance for all ionic species, and the migration fronts of all ions advance deeper into the material. When the water-to-cement ratio increases from 0.30 to 0.40, the penetration front of Na^+^ and the enrichment peak of OH^−^ shift synchronously from 15 mm to 20 mm and 25 mm, respectively, indicating that the increased water-to-cement ratio accelerates the transport rate without altering the electrostatic coupling mechanism between Na^+^ and OH^−^. Meanwhile, the enrichment peak of K^+^, which is driven by the electric field, moves inward, and the height of the Ca^2+^ dissolution peak increases correspondingly. These results demonstrate that a higher water-to-cement ratio enhances the effective diffusion coefficients of all ions, thereby accelerating the inward migration of the ion distribution fronts shaped by both chemical reactions and electrochemical coupling.

### 4.2. Solid Phase Content Distributions

**Figure 19:**
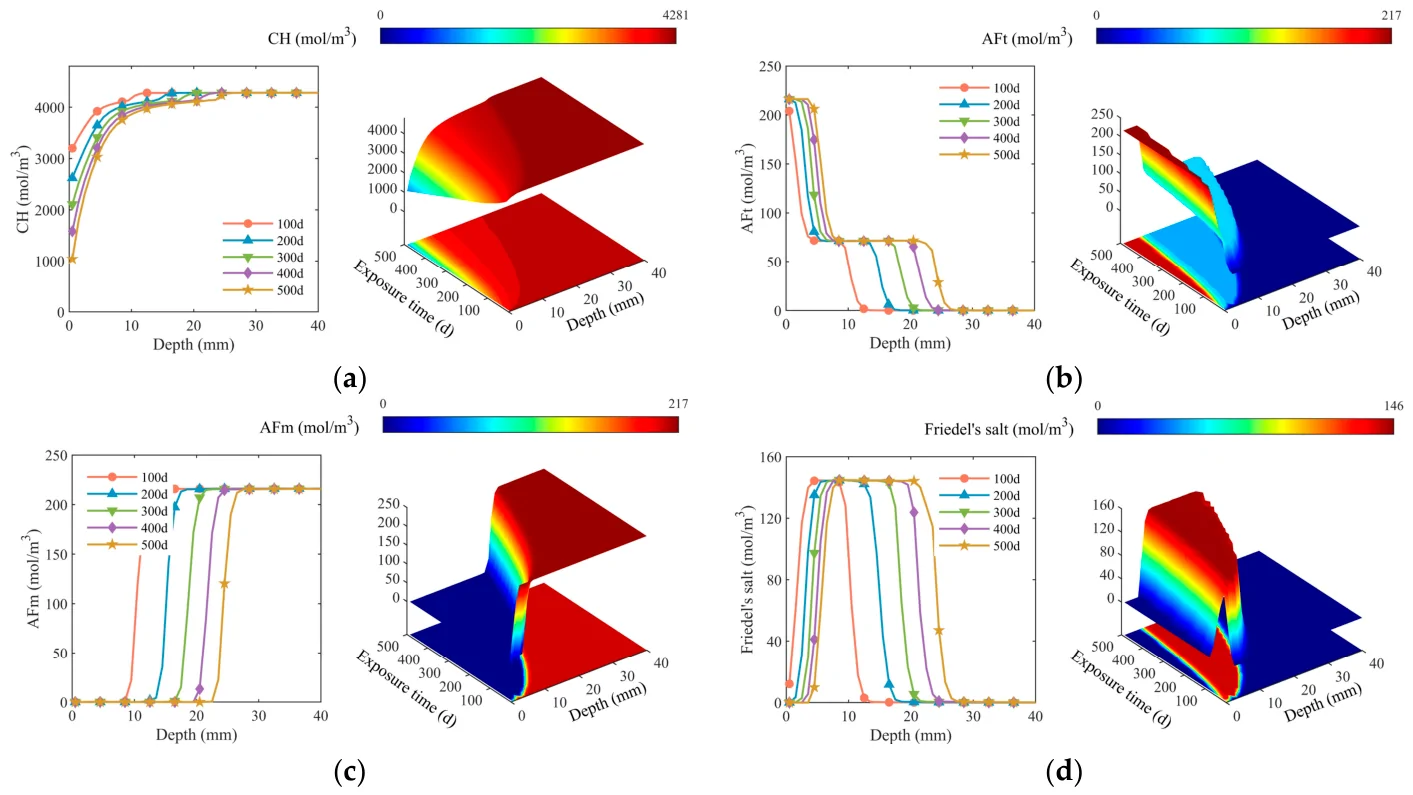
Distributions of solid phase contents under different exposure durations (w/c=0.35): (**a**) CH; (**b**) Aft; (**c**) AFm; (**d**) Friedel’s salt.

Figure 19 presents the spatial evolution of the major solid phases—CH, AFt, AFm, and Friedel’s salt—during exposure from 100 days to 500 days. Under multi-ion coupled attack, the solid phase distributions exhibit clear spatial zonation and chemical coupling. In the surface region, CH undergoes continuous dissolution driven by the concentration gradient and extends deeper with time, accompanied by a progressive decrease in the pH of the pore solution. In the chloride-ingress-dominated zone (e.g., 0–22 mm at 500 days), the initial AFm is completely consumed through anion exchange with Cl^−^ and replaced by a broad Friedel’s salt enrichment plateau, directly reflecting the binding of intruding chloride ions.

Corresponding to the coupled variation in AFm and Friedel’s salt, the spatial distribution of AFt exhibits a characteristic double-step morphology. In the outermost surface layer (0–3 mm), the direct ingress of high-concentration external sulfate produces the highest AFt enrichment peak. As the depth of external sulfate ingress is limited, the AFt content drops rapidly and stabilises at a secondary intermediate plateau. This intermediate plateau results from the decomposition of AFm in the Friedel’s salt enrichment zone, which releases substantial amounts of sulfate and induces secondary AFt precipitation in the interior. Beyond the attack front (approximately 22 mm and deeper), where Cl^−^ has not yet penetrated, AFm remains undecomposed and releases no sulfate; furthermore, external sulfate has not reached this region. Consequently, no additional AFt is generated in the deep zone, and the unreacted original AFm is retained intact in the matrix.

**Figure 20:**
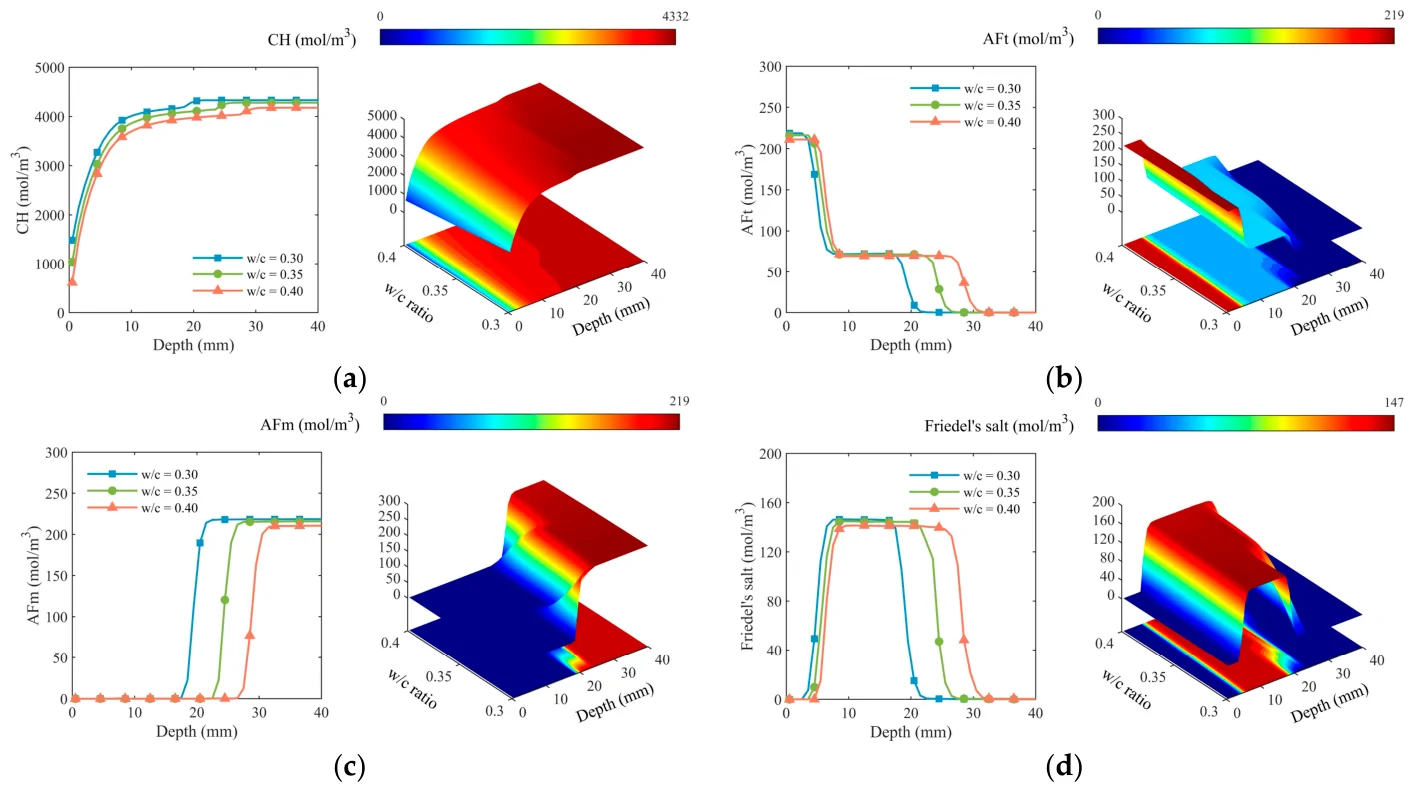
Distributions of solid phase contents under different water-to-cement ratios at 500 days: (**a**) CH; (**b**) Aft; (**c**) AFm; (**d**) Friedel’s salt.

Figure 20 presents the spatial distributions of the solid phase contents for three water-to-cement ratios (0.30, 0.35, and 0.40) at 500 days of exposure. Regarding the initial solid phase inventory, lower water-to-cement ratios correspond to higher solid-phase volume fractions in the hardened cement paste, and the background contents of AFm and CH in the deep unattacked region are correspondingly higher. In terms of the attack process, increasing the water-to-cement ratio significantly raises the initial porosity and pore connectivity, thereby accelerating the diffusion rate of aggressive ions. Consequently, at a water-to-cement ratio of 0.40, the CH dissolution zone, the AFm depletion front, and the enrichment regions of Friedel’s salt and AFt all shift substantially deeper, with the complete consumption front of AFm extending from approximately 19 mm at a water-to-cement ratio of 0.30 to 29 mm at 0.40.

Regarding the absolute concentration levels, the plateau heights of AFt, AFm, and Friedel’s salt exhibit only minor variations among the different water-to-cement ratios. This is mainly because the initial solid phase contents are governed by the hydration reaction equilibrium: although a lower water-to-cement ratio increases the cement content per unit volume, the hydration space constraint also limits the hydration degree, preventing a dramatic increase in the absolute amount of hydration products. Nevertheless, the slightly higher density of C-S-H gel and AFm in the lower water-to-cement ratio paste still provides sufficient reactive sites for the physical adsorption and chemical exchange of ions, ensuring efficient ion immobilisation in the shallow region. On this basis, the more significant role of the water-to-cement ratio is its control over the ion transport rate and the depth of the reaction fronts. The lower water-to-cement ratio, with its denser microstructure and substantially reduced ion diffusion coefficients, confines the physical adsorption, chemical binding, and decalcification reactions to a narrow surface zone, achieving a synergistic protection effect through both physical resistance and chemical interception.

### 4.3. Evolution of Porosity and Microcracks

**Figure 21:**
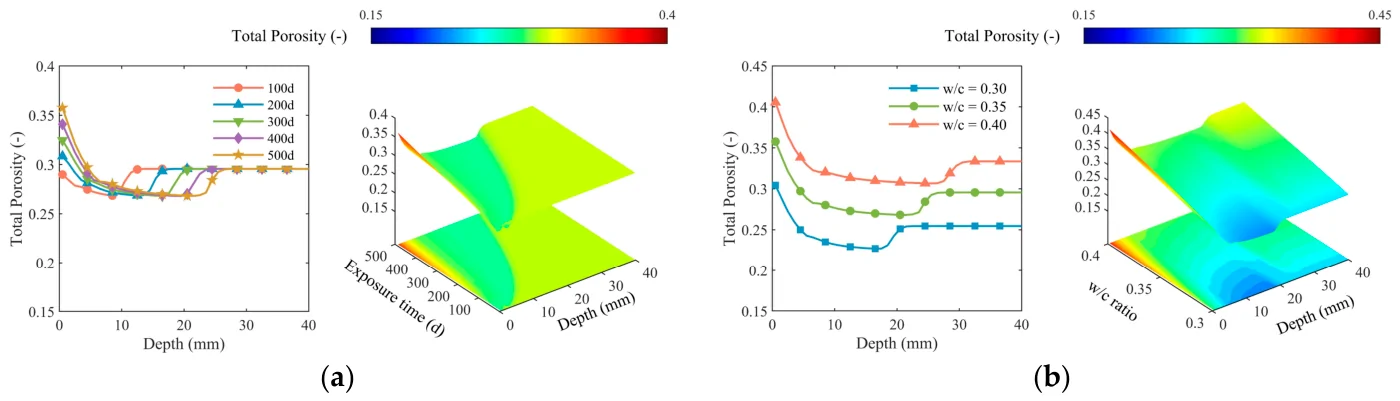
Porosity profiles under different exposure durations and water-to-cement ratios: (**a**) w/c=0.35; (**b**) 250 d.

Figure 21 presents the spatial distribution and evolution of the total porosity within the cement paste under multi-ion attack. As shown in Figure 21a (water-to-cement ratio of 0.35), the porosity profile exhibits a characteristic dual-zone pattern: surface dissolution-induced porosity increase and subsurface precipitation-induced pore filling. With increasing exposure duration, the outermost surface region undergoes progressive CH dissolution and decomposition of hydration products, leading to a continuous and significant increase in porosity as the matrix skeleton is gradually hollowed out. In the subsurface region at a certain depth from the exposed surface, the intruding ions react with the matrix to form Friedel’s salt and ettringite, whose larger molar volumes produce a pore-filling effect that locally reduces the porosity below the original level. As the attack progresses, this dynamic dissolution–filling front advances continuously into the deeper regions.

Figure 21b further reveals the controlling role of the water-to-cement ratio on porosity evolution. As the water-to-cement ratio increases, the initial porosity and pore connectivity of the matrix increase significantly, accelerating the inward diffusion of ions and causing the dissolution-induced porosity increase zone and the precipitation-induced filling zone to extend substantially deeper. It should be noted that the total porosity shown here mainly reflects the matrix porosity evolution caused by solid phase transformations and dissolution, without yet superimposing the crack volume induced by cracking. Although the precipitation of microscopic products temporarily densifies the matrix pores during the early stage of attack, the continued accumulation of crystalline products in confined pores generates a large expansion pressure. Once this expansion stress exceeds the tensile strength of the matrix, microcracks nucleate and propagate. When this additional crack volume is superimposed, the actual total porosity and transport properties of the cement paste undergo more severe secondary degradation at the later stages of attack.

**Figure 22:**
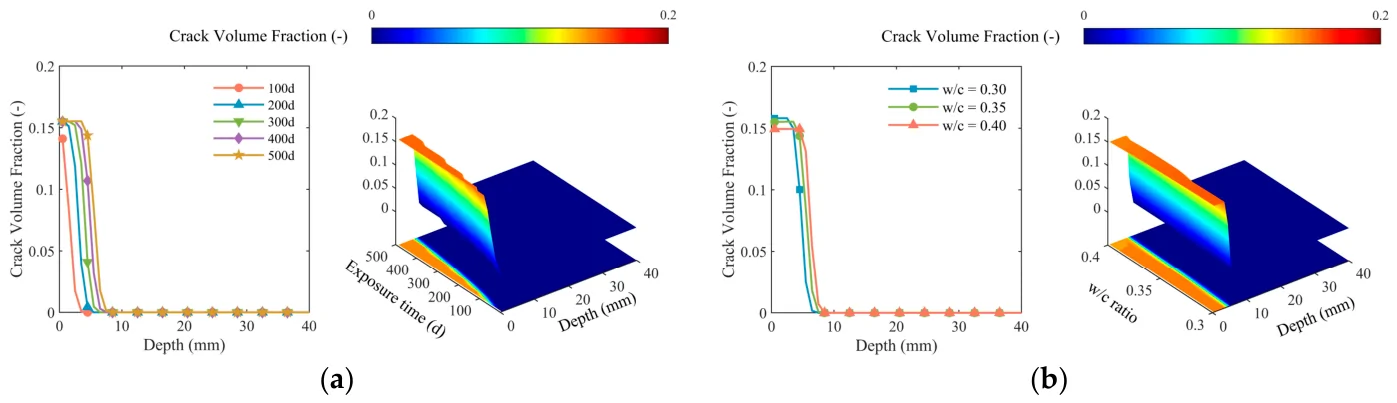
Microcrack volume fraction profiles under different exposure durations and water-to-cement ratios: (**a**) w/c=0.35; (**b**) 250 d.

Figure 22 presents the spatiotemporal evolution of the microcrack volume fraction within the cement paste under multi-ion attack, highlighting the capability of the present model to capture the macro-cracking induced by microscopic crystallization pressure. By coupling microscopic thermodynamic phase transformations with meso-scale damage mechanics, the model achieves a fully coupled simulation of the cracking mechanism: when the ingress of aggressive ions leads to extensive precipitation of expansive corrosion products such as ettringite, and the volumetric expansion within the confined pore space exceeds the accommodation limit, the model automatically triggers the crystallization pressure calculation; once this pressure surpasses the local tensile strength of the matrix, microcrack nucleation is initiated and the crack volume fraction is dynamically computed. As shown in Figure 22a (water-to-cement ratio of 0.35), microcracks are confined to the outermost surface in the early stage of attack. As the exposure duration increases, the continued accumulation of crystalline products in the shallow region causes the cracking damage zone to extend progressively deeper, with the crack volume fraction rising rapidly and eventually reaching a damage saturation level, realistically reproducing the dynamic progression of crystallisation-induced damage from the surface inward. Figure 22b further compares the cracking characteristics under different water-to-cement ratios. The results show that specimens with different water-to-cement ratios all reach a similar damage saturation level in the outermost surface, and the differences in cracking depth are relatively limited. As the water-to-cement ratio increases from 0.30 to 0.40, the slightly faster ion transport under higher water-to-cement ratios causes a modest deepening of the zone in which crystallisation stress accumulates, and the cracking damage front exhibits only a slight inward shift. This indicates that the water-to-cement ratio has a limited influence on the degree of surface cracking, mainly playing a moderating role in the cracking depth to a certain extent, which further confirms that crystallisation-induced damage is predominantly concentrated in the outermost surface medium during the early stage of exposure.

### 4.4. Evolution of the Diffusion Coefficient

**Figure 23:**
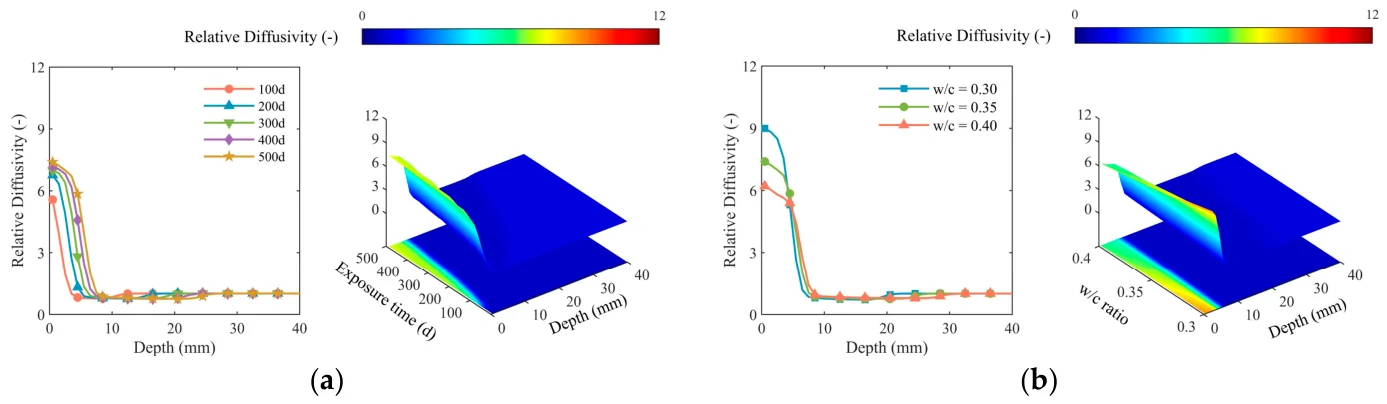
Relative diffusion coefficient profiles under different exposure durations and water-to-cement ratios: (**a**) w/c=0.35; (**b**) 250 d.

Figure 23 presents the spatiotemporal evolution of the relative diffusion coefficient (the ratio of the effective diffusion coefficient to the initial reference value) under multi-ion attack. As shown in Figure 23a (water-to-cement ratio of 0.35), the relative diffusion coefficient profile exhibits a pronounced three-zone differentiation. In the outermost surface region (0–5 mm from the exposed face), massive microcrack nucleation induced by crystallisation pressure causes the relative diffusion coefficient to increase dramatically with time, reaching a value more than seven times the initial value at 500 days, directly revealing the secondary opening effect of cracking damage on ion transport pathways. In the subsurface intermediate region (8–25 mm), the substantial precipitation of solid products with larger molar volumes, such as Friedel’s salt and ettringite, fills the capillary pores and reduces the pore connectivity, causing the relative diffusion coefficient in this region to drop below the initial reference value, reflecting the pore-blocking effect of solid products. In the deep unattacked region, the relative diffusion coefficient remains constant at unity.

Figure 23b further compares the evolution of the relative diffusion coefficient under different water-to-cement ratios, revealing the differential influence of microcracking on matrices with different densities. At 250 days, the relative diffusion coefficient amplification factor in the surface region of the low water-to-cement ratio (0.30) specimen approaches 9, which is significantly higher than the approximately 6 observed for the high water-to-cement ratio (0.40) specimen. The physical mechanism behind this phenomenon lies in the fact that the microstructure of low water-to-cement ratio cement paste is extremely dense, with a very small initial intrinsic diffusion coefficient. When crystallisation-induced cracking occurs in the surface region, the newly formed microcracks provide high-permeability pathways, producing a much more dramatic relative amplification effect in the already dense matrix. This indicates that cracking damage exerts a more severe relative destructive effect on the transport properties of low water-to-cement ratio, high-density cement paste. In the subsurface region, all water-to-cement ratios exhibit a decrease in the relative diffusion coefficient to varying degrees, owing to the precipitation and filling of corrosion products in the capillary pores.

### 4.5. Engineering Implications and Implementation Challenges

The multi-scale transport–reaction framework developed in this study offers a simplified numerical approach to explore the long-term durability of cementitious materials under complex environmental exposure. By integrating multi-species ionic transport with phase transformations, crystallization pressure-induced microcracking, and the resulting staged evolution of diffusion coefficients, the model helps illustrate transport behavior during degradation processes. In practical engineering contexts, this modeling concept can provide a helpful reference for durability assessment and service life estimation across various infrastructure types. For marine structures and bridge piers subjected to combined chloride–sulfate attack, the model can assist in evaluating potential limit states by offering estimates of chloride ingress toward the rebar depth while tracking sulfate crystallization stress to anticipate microcracking risk. Similarly, for nuclear concrete structures and underground containment vaults exposed to long-term groundwater leaching, it can help estimate decalcification progress and dissolution fronts. Furthermore, the observed staged diffusivity evolution provides practical reference for evaluating water-to-cement ratio effects and identifying potential timing for applying surface protective coatings before cracking-induced transport acceleration occurs.

Translating this paste-scale modeling approach into full-scale concrete engineering practice nevertheless involves several open challenges and limitations. Because the present geometric formulation is confined to paste-scale representative volume elements, upscaling to concrete structures requires incorporating coarse aggregates to account for spatial tortuosity and obstruction effects. Additionally, localized porosity variations and transport channels within the interfacial transition zone (ITZ) around aggregates need to be further integrated. Finally, field concrete undergoes ongoing hydration refinement and structural densification over extended exposure durations; incorporating such long-term microstructural aging kinetics remains essential for capturing the evolving baseline transport properties of aging structures. Addressing these mesoscopic and temporal aspects in future work will help advance paste-level modeling toward practical engineering durability evaluation.

## 5. Conclusions

A multi-scale lattice diffusion–reaction coupled framework was established, in which the electrical double layer (EDL) effect is captured through a simplified analytical correction. The spatiotemporal phase evolution and multi-ion transport performance in cement paste under coupled chemical attack were systematically investigated. The main conclusions are drawn as follows.

1. Under multi-ion coupled ingress, hydration products exhibit highly ordered spatial zonation. Ingressing chloride ions completely displace monosulfate (AFm) to form a Friedel’s salt enrichment zone, while the displaced sulfate couples with penetrating external sulfate to trigger secondary ettringite precipitation, generating a characteristic double-step AFt spatial distribution. The depth of the Friedel’s salt enrichment zone and the secondary AFt plateau advances progressively with exposure time, while the surface AFt peak remains confined to the outermost 3–5 mm.
2. When ettringite accumulation exceeds the critical capillary pore filling volume fraction, the excess volume drives microcracking. Combined with calcium-leaching-induced surface porosity increase, this produces a three-zone relative diffusivity profile: a sharp surface rise (exceeding 7-fold at 500 d for w/c = 0.35) from leaching and cracking, a subsurface drop from pore filling by Friedel’s salt and ettringite, and a stable deep baseline. The post-cracking amplification is markedly higher in low-w/c matrices (approximately 9-fold for w/c = 0.30 vs. 6-fold for w/c = 0.40) due to their lower initial diffusivity.
3. The water-to-cement ratio plays a dual role in matrix degradation. A higher w/c ratio accelerates ion ingress and deepens reaction fronts, whereas a lower w/c ratio confines chemical degradation to a narrow surface layer through physical resistance and chemical interception. However, low w/c matrices suffer from a more abrupt relative diffusivity deterioration once microcracking initiates. This dual mechanism highlights that while low w/c cement paste offers superior intrinsic resistance, it demands more proactive crack prevention strategies to sustain long-term performance.
4. By capturing the staged evolution of diffusivity induced by coupled reactions and crystallization-driven microcracking, the proposed multi-scale framework can serve as a quantitative tool to predict the critical time window for applying surface protective coatings and to optimize mixture design. The on-demand phase evolution database strategy enables efficient coupling of thermodynamic equilibrium calculations with multi-scale transport updates, achieving approximately 20-fold computational speedup relative to full RVE recalculation schemes. This database-driven approach makes iterative long-term durability simulations computationally tractable. These findings provide practical implications for durability design and life-cycle management of marine structures, cross-sea bridges, and coastal infrastructure.

While the present framework successfully captures the coupled transport–reaction–damage evolution in cement paste, several inherent assumptions should be acknowledged, including isotropic transport, local thermodynamic equilibrium, saturated isothermal conditions, and simplified RVE crack representations. Future extensions will incorporate non-saturated exposure, freeze–thaw cycling, external mechanical loading, and macro-scale phase-field fracture descriptions to bridge paste-level simulations toward full-scale structural durability assessment.

## Figures and Tables

**Table 1:** Mineralogical composition per unit mass of cement.

Mineral Phase	Mass (g/g Cement)	Moles (mmol/g-Cement)	Molar Mass (g/mol)
$C_{3}S$	0.5796	2.542	228
$C_{2}S$	0.2071	1.204	172
$C_{3}A$	0.0766	0.284	270
$C_{4}AF$	0.0990	0.204	486
$C\bar{S}H_{2}$	0.0377	0.219	172

**Table 2:** Self-diffusion coefficients in bulk water and EDL correction parameters (25 °C).

Ionic Species	$D_{\mathrm{bulk},\;i}$ (10^−9^ m^2^/s)	κ
Cl^−^	2.03	1.0
OH^−^	5.26	1.0
Na^+^	1.33	1.0
K^+^	1.96	1.0
SO_4_^2−^	1.07	0.6
Ca^2+^	0.79	0.6
Al^3+^	0.54	0.4

**Table 3:** Diffusion coefficients of major ions in cement paste (10^−12^ m^2^/s).

w/c	Cl^−^	OH^−^	SO_4_^2−^	Na^+^	K^+^	Ca^2+^	Al^3+^
0.30	2.64	7.45	1.90	2.72	1.35	1.07	0.78
0.35	4.18	10.88	2.79	4.31	2.33	1.77	1.24
0.40	5.79	14.12	4.24	5.66	3.23	2.45	1.78

**Table 4:** Key chemical reactions and thermodynamic equilibrium constants involved in the present model (25 °C).

Reaction Type	Reaction Equation	log $K_{p}$
CH dissolution	$CH+2H^{+}⇌Ca^{2+}+2H_{2}O$	22.80
C-S-H (Jennite-type) dissolution	$C_{1.67}SH_{x}+3.33H^{+}⇌1.67Ca^{2+}+SiO_{2}\left(s\right)+yH_{2}O$	38.00
C-S-H (Tobermorite-type) dissolution	$C_{0.83}SH_{x}+1.67H^{+}⇌0.83Ca^{2+}+SiO_{2}\left(s\right)+yH_{2}O$	15.33
Ettringite (AFt) dissolution	$AFt+4H^{+}⇌6Ca^{2+}+3SO_{4}^{2-}+2Al{\left(OH\right)}_{4}^{-}+H_{2}O$	11.10
Gypsum dissolution	${\mathrm{CaSO}}_{4}\cdot 2H_{2}O⇌Ca^{2+}+SO_{4}^{2-}+2H_{2}O$	−4.58
Monosulfate (AFm) dissolution	$AFm+4H^{+}⇌4Ca^{2+}+SO_{4}^{2-}+2Al{\left(OH\right)}_{4}^{-}+H_{2}O$	26.60
Friedel’s salt dissolution	$FS+4H^{+}⇌4Ca^{2+}+2Cl^{-}+2Al{\left(OH\right)}_{4}^{-}+H_{2}O$	27.00
Kuzel’s salt dissolution	$Kuzel+4H^{+}⇌4Ca^{2+}+0.5SO_{4}^{2-}+Cl^{-}+2Al{\left(OH\right)}_{4}^{-}+H_{2}O$	26.80

## Data Availability

The original contributions presented in this study are included in the article. Further inquiries can be directed to the corresponding author.

## Abbreviations

The following abbreviations are used in this manuscript:

C-S-H	Calcium silicate hydrate
LD C-S-H	Low-density calcium silicate hydrate
HD C-S-H	High-density calcium silicate hydrate
RVE	Representative volume element
EDL	Electrical double layer
AFm	Monosulfate
AFt	Ettringite
CH	Calcium hydroxide (portlandite)
w/c	Water-to-cement ratio
HCSS	Hard core–soft shell
PB	Poisson–Boltzmann
MRE	Mean relative errors
RMSE	Root mean square errors
ITZ	Interfacial transition zone
